# FastEval Parkinsonism: an instant deep learning–assisted video-based online system for Parkinsonian motor symptom evaluation

**DOI:** 10.1038/s41746-024-01022-x

**Published:** 2024-02-08

**Authors:** Yu-Yuan Yang, Ming-Yang Ho, Chung-Hwei Tai, Ruey-Meei Wu, Ming-Che Kuo, Yufeng Jane Tseng

**Affiliations:** 1 Graduate Institute of Biomedical Electronics and Bioinformatics, National Taiwan University, No. 1 Roosevelt Rd. Sec. 4, Taipei, 10617 Taiwan, ROC; 2 Department of Computer Science and Information Engineering, National Taiwan University, No. 1 Roosevelt Rd. Sec. 4, Taipei, 10617 Taiwan, ROC; 3 Department of Neurology, National Taiwan University Hospital, No. 1 Changde St., Zhongzheng Dist., Taipei City, 100229 Taiwan, ROC; 4 Department of Medicine, National Taiwan University Cancer Center, No. 57, Lane 155, Sec. 3, Keelung Rd., Da’an Dist., Taipei City, 106 Taiwan, ROC

**Keywords:** Physical examination, Parkinson's disease, Movement disorders, Software, Machine learning

## Abstract

The Motor Disorder Society’s Unified Parkinson’s Disease Rating Scale (MDS-UPDRS) is designed to assess bradykinesia, the cardinal symptoms of Parkinson’s disease (PD). However, it cannot capture the all-day variability of bradykinesia outside the clinical environment. Here, we introduce FastEval Parkinsonism (https://fastevalp.cmdm.tw/), a deep learning-driven video-based system, providing users to capture keypoints, estimate the severity, and summarize in a report. Leveraging 840 finger-tapping videos from 186 individuals (103 patients with Parkinson’s disease (PD), 24 participants with atypical parkinsonism (APD), 12 elderly with mild parkinsonism signs (MPS), and 47 healthy controls (HCs)), we employ a dilated convolution neural network with two data augmentation techniques. Our model achieves acceptable accuracies (AAC) of 88.0% and 81.5%. The frequency-intensity (FI) value of thumb-index finger distance was indicated as a pivotal hand parameter to quantify the performance. Our model also shows the usability for multi-angle videos, tested in an external database enrolling over 300 PD patients.

## Introduction

The incidence of Parkinson’s disease (PD) increases markedly with age, from 20/100,000 overall to 120/100,000 at age 70^1^ and will be continuously increasing across continents. Parkinsonism is a syndrome of PD that includes resting tremors, rigidity, slowness of movement (bradykinesia), uncontrollable hesitation or interruption in continuous movement, postural instability, and freezing. The Movement Disorder Society PD (MDS-PD) criteria^2^ are developed to guide clinicians in ruling out PD with other atypical parkinsonism (APD). The examination of one of the cardinal manifestations of PD, bradykinesia, is instructed in the MDS-UPDRS part III, the Motor Examination section^3^. This aspect of the clinical evaluation includes finger-tapping, hand open-close movements, and pronation-supination movements, corresponding to items 3.4, 3.5, and 3.6 in MDS-UPDRS part III, respectively.

Although there is a standard guideline for physicians to evaluate the symptoms of Parkinson’s patients some protocol instructions are found to be non-objective. For example, finger-tapping in the MDS-UPDRS is defined as tapping the index finger on the thumb ten times as quickly and as big as possible^3^. Alternatively, a quantitative index, defined as the maximal number of times a person can tap their fingers within 5 s as big and quickly as possible, was used to evaluate the severity of the movement^4^. However, this index does not fully consider the finger taps’ amplitudes and is relatively semi-quantitative. Even though the instructions of this movement have mentioned to do it “as big as possible,” people unintentionally reduce amplitudes to accelerate the speed practically.

Digital biomarkers accessed by artificial intelligence (AI) provide real-time noninvasive monitoring, diagnosing, and treating various medical conditions^5–11^. For example, a smartphone’s accelerometer and gyroscope have been used to examine those with self-reported PD^12^. Consumer cameras combined with skeleton extraction techniques, such as OpenPose^13^, are used to quantify bradykinesia^14–16^. KELVIN^TM^, built by Machine Medicine Technologies^14,17^, and Tencent Medopad^18^ are two recently developed systems for monitoring and examining PD patients’ status. However, current AI-assisted rating systems suffer from two main flaws—in-clinic only monitoring and inherently subjective system leading to inter- and intra-rater variability^19,20^. Furthermore, these systems have not yet addressed the potential bias introduced by variations in camera angles during video recordings. Thus, an objective, at-home, easy-to-use system, capable of accepting multiple camera angles and automatically accessing analyzed digital biomarkers, is worth developing for detecting, monitoring, and evaluating the severity of motor symptoms of PD^21^.

Recently, a number of studies have explored the use of 3D keypoint estimation for quantifying bradykinesia. While these studies offer promising algorithmic advancements, a significant gap remains in their practical application. The majority of these studies have either not released their source code^22,23^ or failed to provide an accessible, web-based system^24,25^. This lack of availability and user-friendliness means that patients and physicians are unable to derive tangible benefits from these tools. Furthermore, while there has been an instance of a study releasing a free online hand analysis system^26^, this solution falls short in terms of functionality. Its stateless design does not allow for the storage of patient records, which is a crucial feature for effective long-term monitoring of bradykinesia. This omission represents a missed opportunity for continuous patient care and hampers the ability of healthcare providers to track the progression of symptoms over time.

Our study aims to delineate an evaluation workflow considering both the speed and amplitude of bradykinesia using a finger-tapping task (FTT). The video-based dataset was collected from patients and healthy subjects for deep-learning model training. Digital biomarkers are accessed by a 3D keypoint extractor, MediaPipe^27^, combined with two data augmentation methods—3D keypoint rotation and video random cropping to overcome the bias induced by the variations in camera angles. The AI-estimated MDS-UPDRS item scores were compared with traditional evaluating indices (using frequency and intensity) in a validation cohort with a public PD motor dataset (PDMotorDB)^4^. Finally, to achieve the goals of the home-based system, we built up a website for the self-evaluation and remote long-term monitoring of hand movements.

## Results

### Participant characteristics

**Fig. 1:**
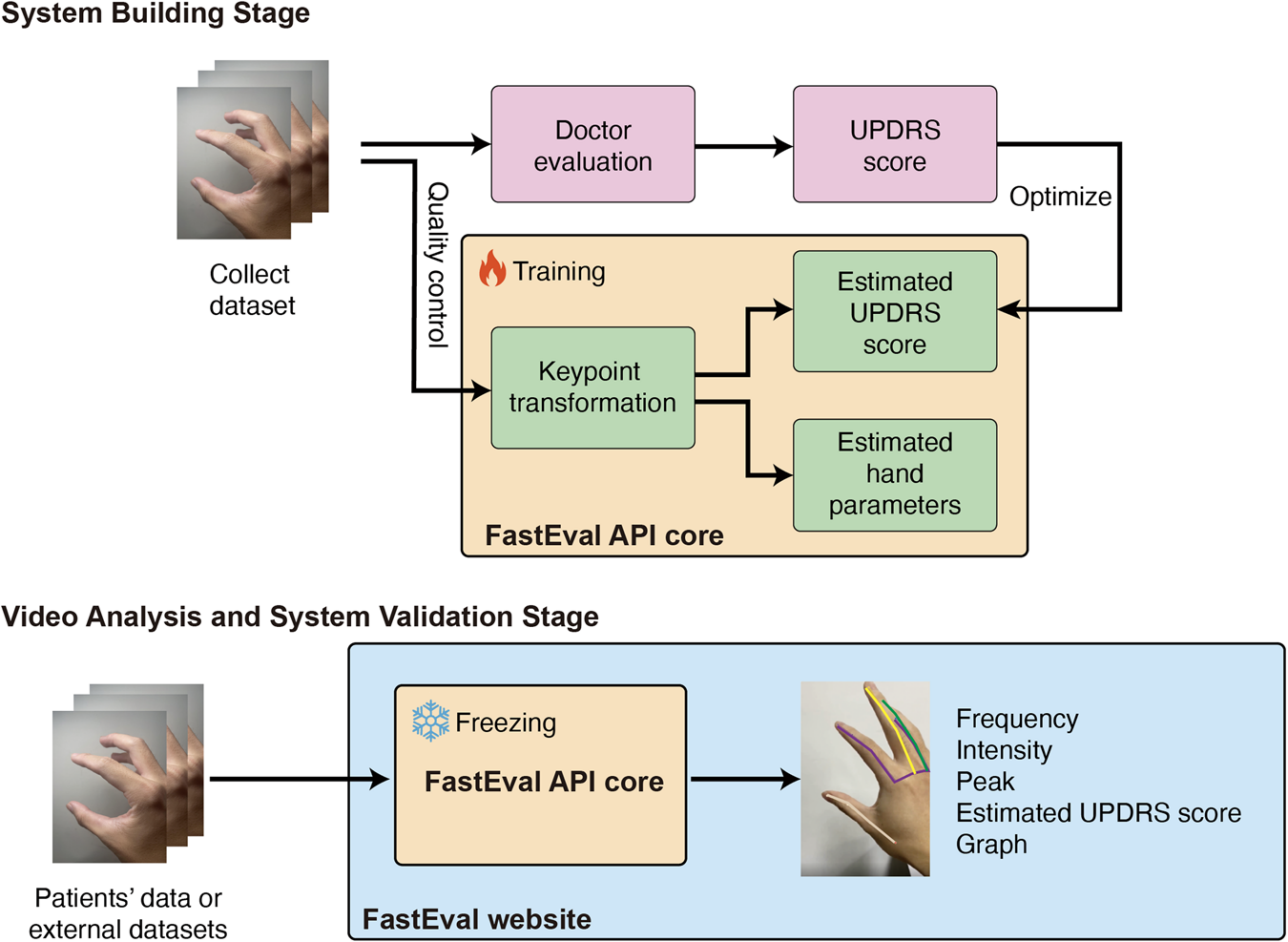
Overview of workflows for system-building, validation, and video analysis. For the system-building stage, each clip was scored independently by a movement disorder specialist. At the same time, the hand skeleton in each clip was extracted using a combination of MediaPipe and preprocessing methods (including normalization and null value processing) to ensure the quality of the hand keypoints for model training and testing. Then, the hand keypoints can be used in deep learning model building and quantitative hand parameters calculation. The hyperparameters were optimized by grid-searching and data augmentation (3D keypoint rotation and random cropping). A well-trained model was picked to estimate the MDS-UPDRS item score. Furthermore, four hand parameters were calculated and compared with the estimated MDS-UPDRS item score to verify and interpret the model. For the video-analyzing stage, an inferencing pipeline was built, including the keypoint transformation, quantitative hand parameters calculation and MDS-UPDRS item score estimation. The model was also verified by an outer validation dataset. Lastly, users can access the service to assess their motor movement via the website interface.

210 patients’ visits from 186 participants (103 PD, 24 participants with APD, 47 healthy controls (HCs), 12 elderly with mild parkinsonism signs (MPS)^28,29^) were enrolled from one community-based populations and two hospital-based cohorts from National Taiwan University Hospital (NTUH) and National Taiwan University Cancer Center (NTUCC) between October 19, 2020, and August 31, 2022. APD are a group of heterogeneous neurological degenerative diseases with bilateral parkinsonism, such as multiple system atrophy (MSA). A total of 840 video clips recording finger-tapping for each hand separately were analyzed. Figure 1 shows the workflows in this study. Due to the clinical characteristics of APD, this group had a lower age and higher male ratio compared to the other three groups (Supplementary Table 1).

To ensure accuracy in data assessment, the diagnosis and motor scores for each patient were primarily evaluated independently by a single movement disorder specialist (Dr. Ming-Che Kuo). Additionally, to further validate these assessments, an extra evaluator was involved, and the evaluations are detailed in Supplementary Table 2, Supplementary Table 3, and Supplementary Table 4. However, to avoid inter-rater discrepancies and maintain consistency in our analysis, only the labels from Dr. Kuo were utilized in the final dataset. Finger-tapping clip scores for each hand were averaged for each patient’s visit unless the symptom asymmetry was investigated. However, due to limited data in the MDS-UPDRS item scores of 3 and 4, we combined two groups into a single group (score of 3+) when training deep-learning models.

HCs typically had no clinical symptoms and were scored 0, but some, due to natural aging, received a score of 1 for slower finger tap performance (Supplementary Fig. 1). Participants in the MPS group are those with MPS^28,29^, but did not meet the diagnostic criteria of PD^2^ or MSA^30^, a subtype of APD. A small amount of them reduced their hand movement functions; therefore, their scores ranged from 0 to 1. The MDS-UPDRS scores of finger-tapping during each subject’s visit showed more severe symptoms in both hands for APD participants than typical PD patients (Supplementary Table 1). Our findings indicate symmetrical hand movement impairment in most APD participants, while PD patients tend to have a more asymmetrical impairment with a high ratio of asymmetry to symmetry (0.40).

### Data quality control

We trained and tested the models with a dataset having a cutoff threshold of the error frames ratio (TEFR) of 0.3 or 0.5. The selection of these two TEFRs was based on our data distribution, with 75% and 85% of clips achieving these respective TEFRs. Given the number of available clips, we opted not to utilize a TEFR below 0.3. The results implied that the low TEFR would slightly reduce the number of utilizable videos and further lessen model performance (Supplementary Table 5). However, based on two-tailed Student’s *t*-test (*α* = 0.05), there was only marginal difference (*p* > 0.05). Namely, the TEFR had minimal effect within a small range. For broader applicability, it was fixed at 0.5 for following model training.

### Training and tuning MDS-UPDRS item score subtasks with binary classifiers

The performance of three architectures indicates that the original PDHandNet by Ho^31^ and multichannel convolutional neural network with the gated recurrent units (CNN-GRU) achieved a higher validation Matthews correlation coefficient (MCC) (0.38) compared to the modified PDHandNet (Supplementary Table 6). The original PDHandNet and the multichannel CNN-GRU model by Lu et al.^32^ had apparent advantages in our classification subtasks. In this study, the original PDHandNet was chosen as the main neural network architecture because of its applicability and computing efficiency^31^.

The hyperparameter grid-search experiments demonstrate that using a large batch size in the training of binary classifiers might constrain the model to a specific local minimum in binary classification tasks (see Supplementary Fig. 2). A larger learning rate allows the model for faster convergence (Supplementary Fig. 3), while L2 regularization^33^ was added in the objective function to prevent potential overfitting. Thus, we chose a smaller batch size and a larger learning rate with L2 regularization policy in our model training stage.

### Estimation of MDS-UPDRS item score with 3D keypoint rotation

When we introduced 3D keypoint rotation augmentation during the model-picking stage, it had a detrimental effect on validation performance, as shown in Supplementary Table 7. Specifically, the validation and testing MCC for a model with 3D keypoint rotation at training and model-picking stage (Model-w-3D-tp) were 0.60 and 0.28, respectively, which were lower than the corresponding MCCs of 0.66 and 0.38 achieved by a model with 3D keypoint rotation merely at training stage (Model-w-3D-t). Similarly, for a model with 3D keypoint rotation at training, model-picking, and inference stage (Model-w-3D-tpi), the validation and testing MCC scores were 0.60 and 0.50, respectively, while a model with 3D keypoint rotation at training and inference stage (Model-w-3D-ti) achieved MCCs of 0.66 and 0.56. Upon closer examination of the models with the testing MCC (Model-w-3D-tp versus Model-w-3D-tpi or Model-w-3D-t versus Model-w-3D-ti), we observed a general improvement, typically by approximately 0.20, after implementing 3D keypoint rotation at the inference stage.

**Fig. 2:**
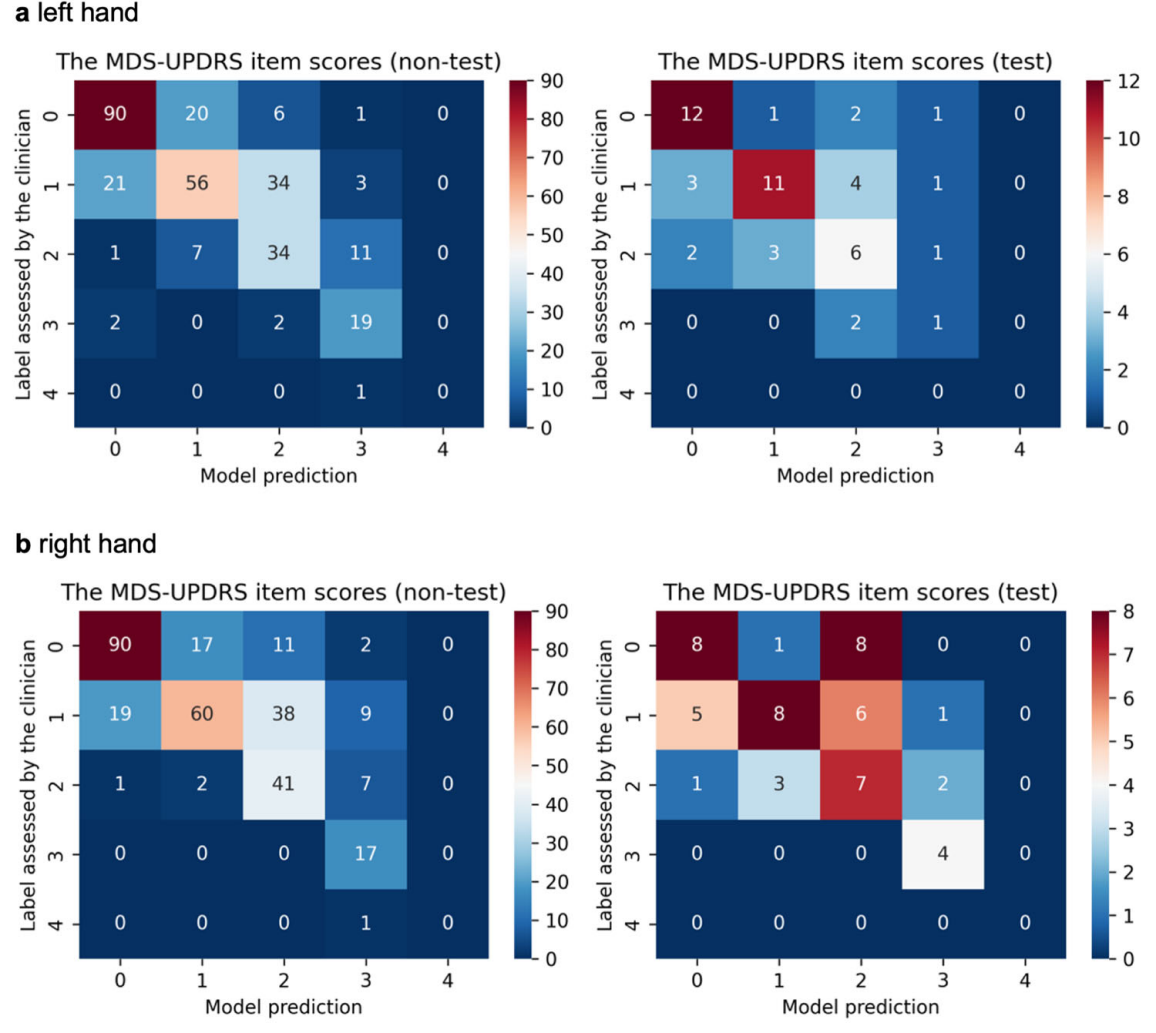
Confusion matrix of the MDS-UPDRS item scores assessed by the clinician and the best-selected model (Model-w-3D-tpi) for the left and right-hand finger tapping task in non-testing and testing dataset. **a** Left-hand finger tapping task; **b** right-hand finger tapping task. The numbers in the confusion matrix represent the count of files. These results indicate that the estimations were generally reasonable and accurate, with only a few exceptions.

When evaluating the performance of our models on the multiple-label task, we noted that the most successful model for estimating left-hand MDS-UPDRS item scores was Model-w-3D-tpi, with a high acceptable accuracy (AAC) of 88.0% and a Cohen’s kappa coefficient (Kappa) of 0.433 (Supplementary Table 8). The confusion matrix, which serves as a prediction summary for classification and illustrates both misclassifications and perfect predictions^34^, indicates that the estimations were generally reasonable and accurate with only a few exceptions (Fig. 2). For right-hand finger-tapping testing videos, Model-w-3D-ti (AAC = 85.2%, Kappa = 0.281) and Model-w-3D-tpi (AAC = 81.5%, Kappa = 0.381) performed better among all the models (Supplementary Table 8). The confusion matrices of Model-w-3D-tpi- and Model-w-3D-ti-estimated scores show that the models tended to overestimate MDS-UPDRS item scores (Fig. 2 and Supplementary Fig. 4).

### Demonstration of hand parameters

**Fig. 3:**
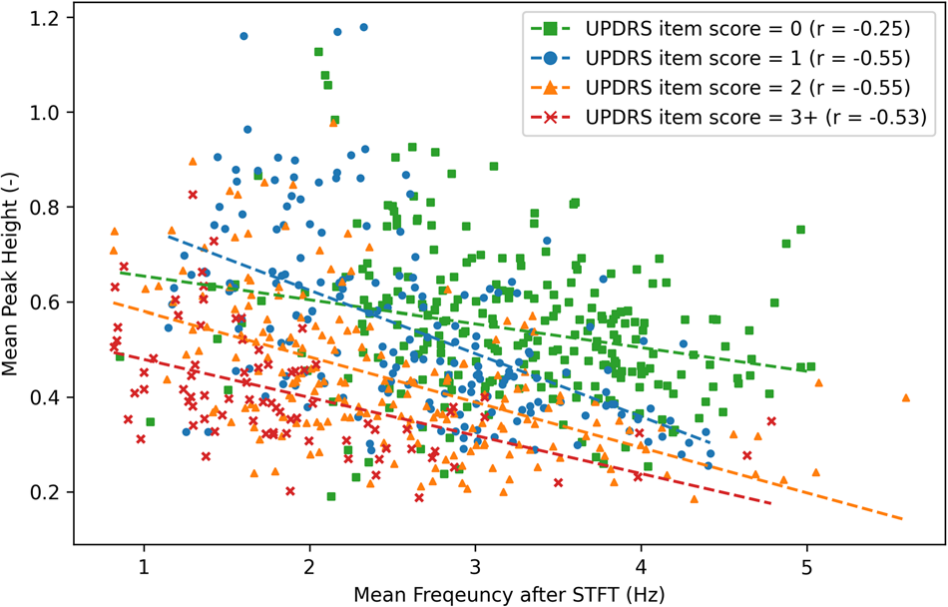
Relationship of the averaged frequency and peak for each clip of finger taps for left and right hands in our dataset. Each time-series dataset was subjected to averaging, presenting an overall depiction of the motor movement status for each recording. The correlation between the averaged frequency and peak was exhibited, showcasing an escalating severity alongside a decline in both frequency and peak. Moreover, the negative correlation coefficient signifies the importance of simultaneously assessing patients’ movement speed and amplitude.

The peak and the intensity in our dataset positively correlated with an overall coefficient of determination (*R*^2^) of 0.784 (Supplementary Fig. 5). Notably, the arbitrary intensity from the short-time Fourier transform (STFT) is associated with practical length. Comparing HCs with patients having different MDS-UPDRS item scores, an analysis of four hand parameters revealed differences in frequency, intensity, frequency-intensity value (FI value or intensity rate), and peak. Specifically, participants with impairments scored 2 or 3+ showcased consistently lower values across all four parameters in contrast to relatively healthier or slightly impaired individuals (Supplementary Table 9). The frequencies for scores 1 and 2 were indistinguishable, while the intensity and peak patterns grouped scores 0 and 1 with scores 2 and 3+. Furthermore, we observed a negative correlation between frequency and peak (or intensity) (Fig. 3). This observation reinforced our proposition that no singular parameter can holistically capture the clinical nuances of hand movements, inspiring the concept of AI-assisted presentation and concurrent analysis of multiple hand parameters on our website.

**Fig. 4:**
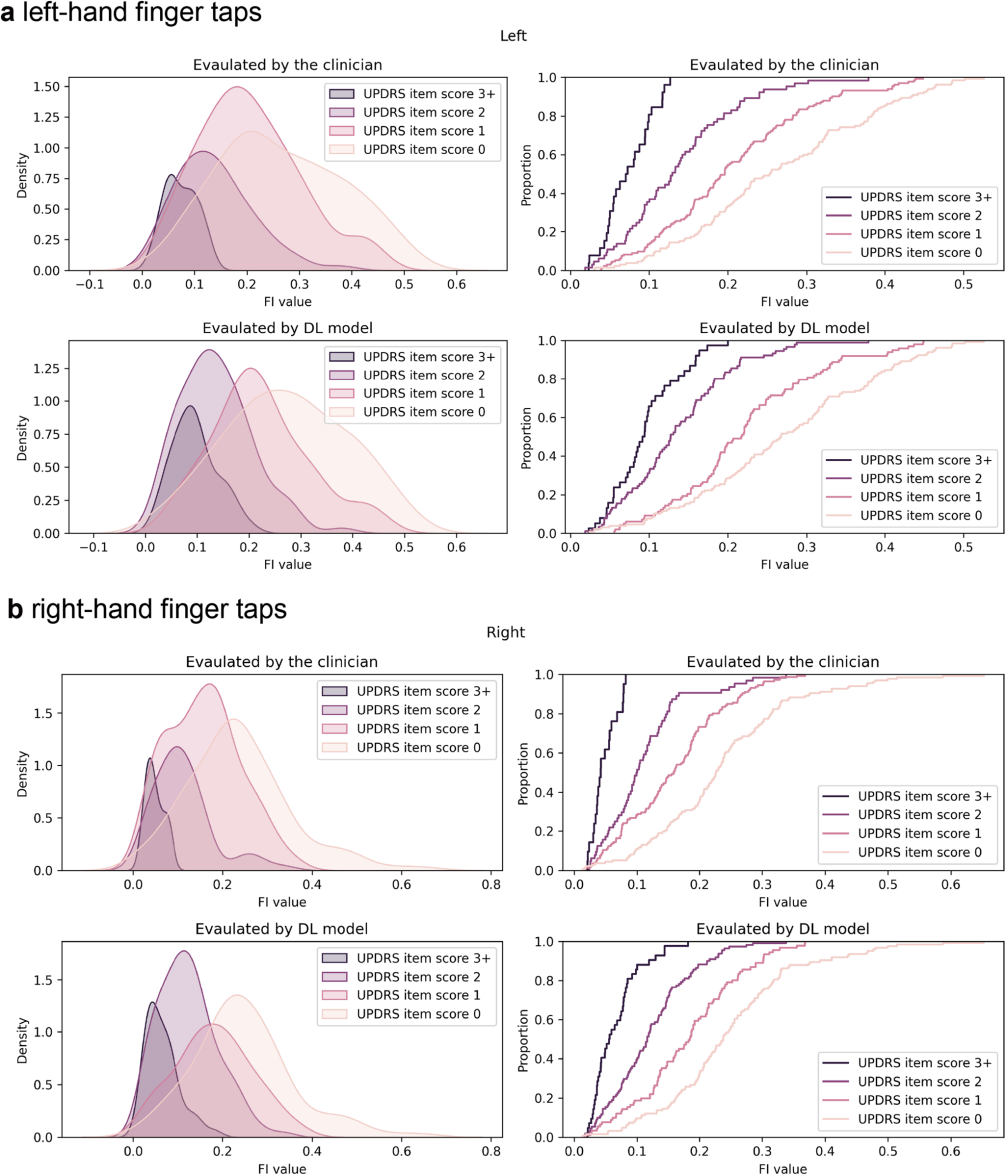
Distribution and the cumulative percentage of the averaged FI value in each MDS-UPDRS item scored by the clinician and Model-w-3D-tpi in our dataset. **a** Left-hand finger taps; **b** right-hand finger taps. To facilitate hand parameter comparison between files, we averaged the time-series FI value to depict its characteristics. We utilized kernel density estimation to visualize the distribution within each score group. On the *x*-axis, you can find the FI values for each score group, while the corresponding number of values is expressed as a density on the *y*-axis. Notably, the MDS-UPDRS item scores serve as indicators of motor movement severity, exhibiting a strong correlation with the averaged FI value. A higher FI value signifies an individual’s proficient performance in finger taps during recording. Furthermore, the cumulative percentage plot unveils the distinctions among score groups while also highlighting the median and quantile within each group. Remarkably, the cumulative percentage plot pattern for each MDS-UPDRS item estimated by our model closely mirrors that of each MDS-UPDRS item scored by the clinician. Furthermore, the FI value (arbitrary units per second, A.U./s) can be converted to a frequency-peak value (thumb lengths per second, thumb-length/s) using the formula provided in Supplementary Fig. 5. For instance, the median FI value (as depicted in Table 1) for individuals with an MDS-UPDRS item score of 0 for left hand is 0.267 (A.U./s), which corresponds to a converted value of 1.42 (thumb-length/s). Similarly, in the case of individuals with an MDSUPDRS item score of 3 for left hand, the value can be converted from 0.042 (A.U./s) to 0.35 (thumb-length/s).

Among these hand parameters, the FI value emerged as the most discriminative one for distinguishing scores, effectively encapsulating the speed of finger-tapping while duly accounting for the intricate relationship between frequency and amplitude (Fig. 4). The medians of the FI value in each group are separated and increase with the decaying MDS-UPDRS item score. This finding suggests that the FI value serves as a comprehensive indicator of motor function, quantitating how big and quick the movement is within a single index and offering valuable insights into the clinical assessment of finger-tapping proficiency.

Furthermore, based on the conversion from intensity to peak (as depicted in Fig. 4 and Supplementary Fig. 5 and Table 1), a subject with an MDS-UPDRS item score of 0 moved their thumb and index finger at a rate of approximately 1.4 thumb lengths per second for the left hand and 1.3 thumb lengths per second for the right-hand. In contrast, a patient with an MDS-UPDRS item score of 3+ could only move their fingers at a rate of approximately 0.4 thumb lengths per second for both hands. Our conversion approach provides a practical way to interpret and relate the FI values to real-world lengths and speed, thus quantitating finger-tapping performance.

**Table 1:** Hand parameters statistics in each score group in our cohort dataset (MDS-UPDRS item scored by Model-w-3D-tpi)

Hand side	MDS-UPDRS	Frequency (Hz)		Intensity (arbitrary unit, A.U.)		FI value (A.U./s)		Peak (distance/thumb-length)	
	item score	Average	Median	Average	Median	Average	Median	Average	Median
Left-hand finger taps	0	3.203 ± 0.653	3.211	0.086 ± 0.039	0.082	0.272 ± 0.117	0.267	0.546 ± 0.161	0.525
	1	2.750 ± 0.783	2.635	0.089 ± 0.048	0.074	0.222 ± 0.093	0.218	0.561 ± 0.209	0.516
	2	2.310 ± 0.766	2.170	0.066 ± 0.040	0.059	0.136 ± 0.069	0.069	0.459 ± 0.173	0.431
	3+	1.928 ± 0.779	1.775	0.058 ± 0.031	0.048	0.096 ± 0.042	0.042	0.417 ± 0.132	0.397
Right-hand finger taps	0	3.255 ± 0.874	3.206	0.078 ± 0.033	0.076	0.247 ± 0.112	0.238	0.539 ± 0.143	0.531
	1	2.452 ± 0.764	2.440	0.080 ± 0.044	0.074	0.181 ± 0.084	0.185	0.525 ± 0.161	0.475
	2	2.623 ± 0.942	2.366	0.054 ± 0.032	0.044	0.122 ± 0.065	0.116	0.420 ± 0.135	0.392
	3+	1.867 ± 0.854	1.707	0.043 ± 0.028	0.031	0.065 ± 0.037	0.056	0.401 ± 0.120	0.366

### Leveraging 3D keypoint rotation for estimating scores of multi-angle videos with external data

We validated our model by using an external PD motor dataset (PDMotorDB) provided by Yang et al. ^4^ With the original labels, the results demonstrated unexpectedly low accuracy (left: AAC = 75.6%, Kappa = 0.058; right: AAC = 86.8%, Kappa = 0.087) compared to our cohort dataset (left: AAC = 88.0%, Kappa = 0.433; right: AAC = 81.5%, Kappa = 0.318) using Model-w-3D-tpi (Supplementary Table 8 and Supplementary Table 10). To investigate the cause and how the model estimated the MDS-UPDRS item scores, we examined the hand parameters for each MDS-UPDRS group in the PDMotorDB dataset.

**Fig. 5:**
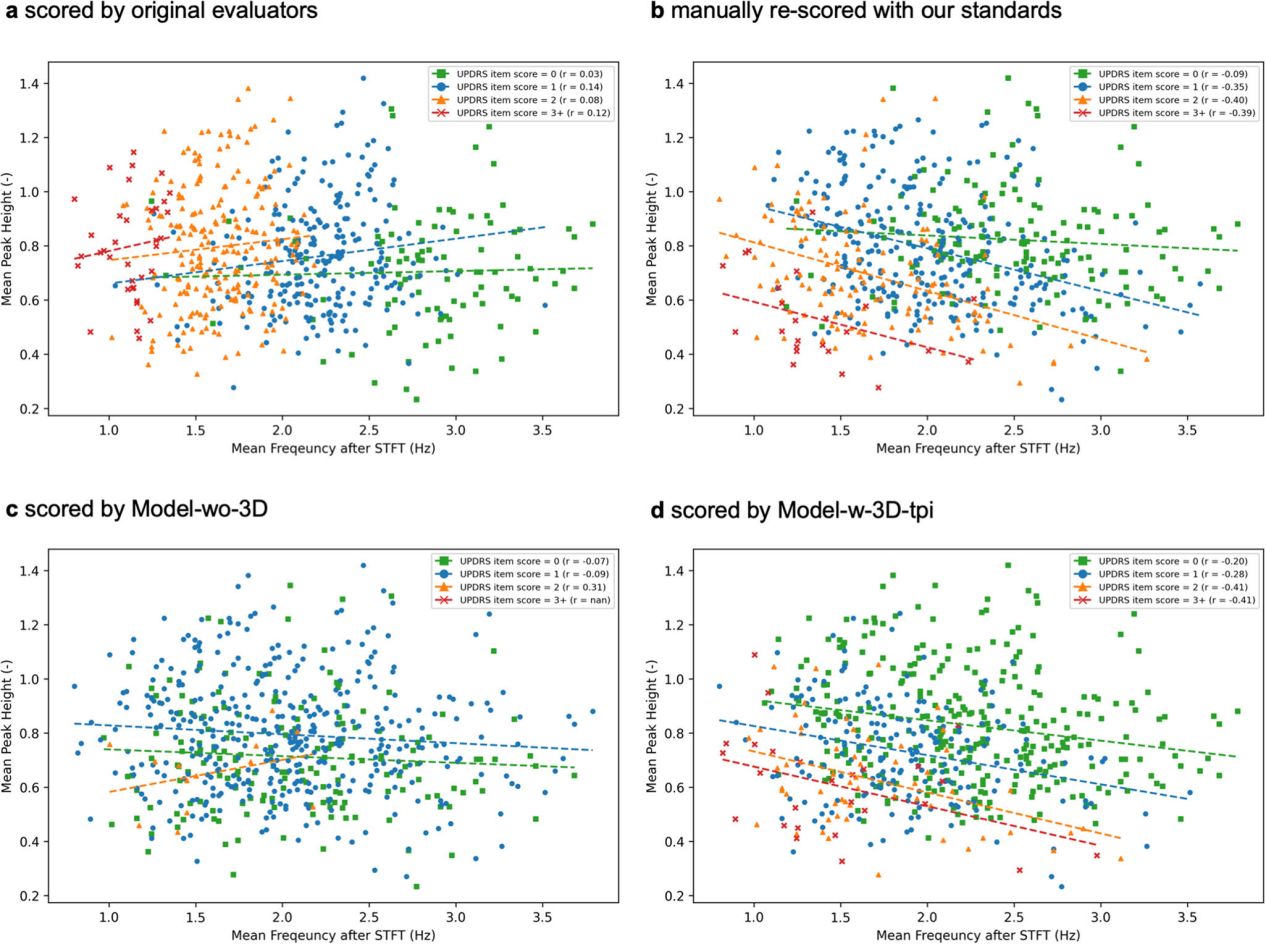
Relationship between the peak and frequency in each MDS-UPDRS group for both hands in the PDMotorDB dataset. All four plots elucidate the associations between averaged peak and averaged frequency, derived from short-time Fourier transform (STFT), within each MDS-UPDRS group, while MDS-UPDRS item scores were appraised by different assessors. **a** The MDS-UPDRS item was evaluated by original evaluators. The primary assessment criterion is the frequency of finger taps, with rough boundary values set at 1.25, 2.0, and 2.5 Hz. **b** The MDS-UPDRS item was re-evaluated following our criteria, revealing a trend of severity growth with decreased peak and frequency. **c** The MDS-UPDRS item was evaluated by Model-wo-3D. Absent the 3D keypoint rotation, the model encountered challenges in estimating accurate scores for frontal-view videos, thereby struggling to exhibit the pattern of severity growth aligned with decreased peak and frequency. Additionally, no estimation was provided for a score of 3. **d** The MDS-UPDRS item was assessed by Model-w-3D-tpi. Benefitting from 3D keypoint rotation techniques during training, model selection, and inference stages, this model mirrors a similar decision-making approach to that of the clinician’s evaluation. The trend of severity growth with decreased peak and frequency is notably preserved.

First, we revealed an easily overlooked aspect of movement assessment by exploring the relationship between frequency and peak. Specifically, upon comparing the assessments of the original evaluators with those generated by our deep learning model, it became apparent that the former placed significant emphasis on frequency as a primary determinant of scores (Fig. 5a, d). Our clinical observations further underscored that individuals naturally tend to exhibit subtle movements during rapid actions. Taking this into consideration, we advocate for a comprehensive evaluation of motor movements that simultaneously considers both frequency and intensity, or peak, in order to provide a more precise assessment. Figure 5b, d demonstrated that the scores based on our clinician’s standards exhibit a similar tendency regarding frequency and peak to those estimated by Model-w-3D-tpi.

Remarkably, whether employing the original labels or scores forecasted by our proficiently trained Model-w-3D-tpi, the FI value consistently emerges as a continuous representative hand parameter, offering clinical insight into the interplay between motor movement speed and amplitude. This correlation is substantiated by the PDMotorDB dataset (Supplementary Fig. 6). The median FI value in each score group decreases with the growth of the MDS-UPDRS item score, which means that the clinician can quantify the severity with more than four levels mentioned in the MDS-UPDRS guideline. It can solve the ambiguousness of the severity between scores.

Furthermore, it has come to our attention that the orientations of recordings in PDMotorDB differ from those in our collected dataset, with the former employing a frontal visual angle. This discrepancy becomes apparent in Fig. 5c, highlighting the challenge that arises in precisely assessing the severity of scores using a model lacking 3D keypoint rotation. In contrast, Model-w-3D-tpi demonstrates resilience to clips from varying perspectives and exhibits a consistent tendency between the internal and external validation datasets (Fig. 5d).

Lastly, the statistical analysis of hand parameters within each score group, estimated and classified by Model-w-3D-tpi, is presented in Table 1 for our cohort dataset and Table 2 for the PDMotorDB dataset. From a clinical standpoint, it was observable that a healthy individual can execute rapid and expansive movements^35^, whereas individuals with parkinsonism faced difficulties in their execution, wherein the severity of their condition directly influenced the extent of their struggle. Notably, four hand parameters exhibit a discernible pattern that correlates with the MDS-UPDRS item score in both our dataset and PDMotorDB dataset. However, there exists a discrepancy in the quantities between these two datasets. The average and median frequencies in each subgroup of item scores within the PDMotorDB dataset were lower than those in our cohort dataset. In contrast, the intensity and peak values in the former dataset were relatively higher. It is also worth mentioning that the FI value remained consistent across the datasets. Further elaboration on this observation will be provided in the discussion section.

**Table 2:** Hand parameters statistics in each score group in the PDMotorDB dataset (MDS-UPDRS item scored by Model-w-3D-tpi)

Hand side	MDS-UPDRS	Frequency (Hz)		Intensity (arbitrary unit, A.U.)		FI value (A.U./s)		Peak (distance/thumb-length)	
	item score	Average	Median	Average	Median	Average	Median	Average	Median
Left-hand finger taps	0	2.207 ± 0.571	2.210	0.126 ± 0.045	0.122	0.271 ± 0.118	0.255	0.807 ± 0.203	0.765
	1	1.743 ± 0.426	1.731	0.108 ± 0.047	0.105	0.180 ± 0.084	0.175	0.695 ± 0.193	0.678
	2	1.624 ± 0.461	1.528	0.091 ± 0.054	0.080	0.134 ± 0.078	0.126	0.633 ± 0.202	0.597
	3+	1.413 ± 0.455	1.246	0.080 ± 0.024	0.078	0.107 ± 0.033	0.094	0.494 ± 0.138	0.483
Right-hand finger taps	0	2.327 ± 0.539	2.269	0.137 ± 0.046	0.134	0.316 ± 0.124	0.301	0.882 ± 0.212	0.862
	1	1.993 ± 0.476	1.934	0.121 ± 0.042	0.120	0.237 ± 0.090	0.232	0.763 ± 0.166	0.765
	2	1.783 ± 0.520	1.593	0.082 ± 0.037	0.082	0.127 ± 0.053	0.129	0.619 ± 0.149	0.593
	3+	1.534 ± 0.546	1.459	0.074 ± 0.039	0.063	0.093 ± 0.041	0.090	0.665 ± 0.180	0.666

### Functionality of FastEval Parkinsonism

FastEval Parkinsonism is publicly available at https://fastevalp.cmdm.tw/, which has three main features: (1) multiple evaluation indices for hand movements, (2) symmetric comparison of hand movements, and (3) monitoring the status of hand movements. All users must register an account to access the web services. After signing up for an account, users can log in to the system. For accurate results, it is recommended that the uploaded side-view video meets certain criteria: exceeding 5 s, 720p resolution, and 60 frames per second. Videos that do not meet these criteria may have less reliable results, even after automatic conversion to the format for the Hand Predictor application programming interface (API). The “Record at” column records the user-defined timestamp of the recording. The end-to-end estimated MDS-UPDRS item score and keypoint-based confidence of the estimation are shown in respective columns.

**Fig. 6:**
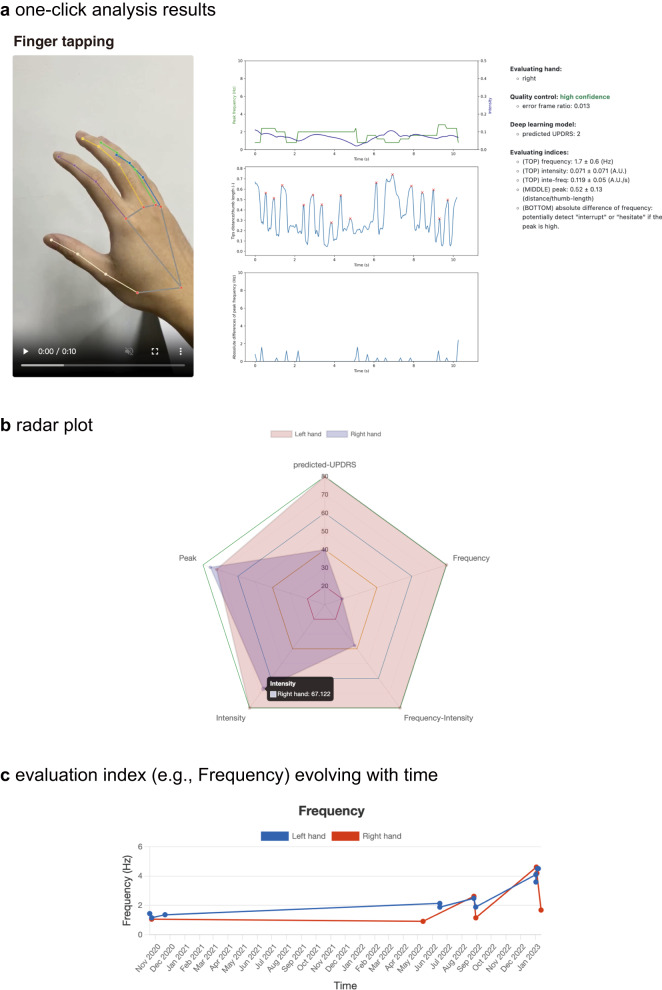
Examples from the FastEval Parkinsonism system. **a** The illustration depicts the outcome of a one-click analysis. Keypoint skeletons were generated using MediaPipe to annotate the provided video. In the upper middle position, the evolving frequency and intensity over time are showcased, effectively capturing the motor movement dynamics during recording. In the middle, the normalized distances between the index finger’s tip and the thumb tip are illustrated, with annotated detected peaks. The middle’s bottom plot exhibits the absolute frequency difference, serving as a potential indicator of interruptions or hesitations. This is due to the noticeable frequency change in case of motor movement interruptions. On the right panel, additional digital details are presented, encompassing the assessed hand, confidence level, estimated MDS-UPDRS item score, and hand parameters (evaluation indices). **b** The showcased radar plot serves as a clear example, vividly depicting the contrast in motor movement severity between the left and right hands. This distinction is achieved by employing four distinct hand parameters alongside a label estimated by a deep-learning model. Notably, both the hand parameters and the label undergo linear transformation to an 80–20 scale, using the median of our cohort dataset as the reference point. This visualization, displaying both hands simultaneously, proves instrumental for clinicians in conducting a rigorous quantitative evaluation of severity. Additionally, it holds the potential to facilitate the early diagnosis of atypical parkinsonism cases. **c** This example offers a representative illustration of time-dependent tracking on a finger-tapping hand parameter, frequency. Through this visualization, clinicians can effectively assess the progression between two clinical visits and potentially adjust medication dosages to enhance the efficacy of treatment plans.

The FastEval Parkinsonism system offers multiple evaluation indices for hand movements. The uploaded video is analyzed using the Hand Predictor API. An example of the FTT results is shown in Fig. 6a. The user determines the evaluating hand’s side, followed by file quality control. The deep learning model, Model-w-3D-tpi, estimates the MDS-UPDRS item score. Scores of 4 are excluded due to insufficient data and ease of assessment. The evaluating indices, including frequency, intensity, FI value, and peak, are displayed. A radar plot is provided to the users to visually compare the left and right-hand clinical features. The scores and hand parameters are linearly transformed to the 80-20 scoring scale for comparison by using the medians of each hand parameter in Table 1. The radar plot is only shown when both left-hand and right-hand videos are uploaded, displaying the latest records based on the timestamp (Fig. 6b). In this example, the right-hand movement is poorer than the left hand, and the symptoms were observed in the frequency, with a score of 20 on the 80–20 scale for the right-hand. Considering the speed and amplitude of the movement simultaneously, the right-hand FI value showed a similar level compared to the estimated MDS-UPDRS item score. Thus, the individual demonstrated in Fig. 6b is indicated to have right-side dominant bradykinesia. By integrating self-assessment capabilities for patients, we empower them to actively participate in their health monitoring process (Fig. 6c).

## Discussion

The discrepancies in intensity, peak values, and smaller frequencies in the PDMotorDB dataset are primarily due to the normalization technique used, involving thumb-length as a standard for hand size. This method caused misconceptions because of shorter thumb lengths extracted by MediaPipe in front-view videos, leading to potential underestimation of severity when relying solely on intensity and peak values. Furthermore, the underestimation of frequency and consistent FI value is linked to our deep learning model’s estimating mechanism, which inherently estimates the severity and incorporates multiple features, including peak, intensity, and frequency. The FI value, however, is strongly associated with the severity, as mentioned in the result section. The interconnected nature of the FI value formula components meant that overestimating intensity led to underestimating frequency. Nonetheless, through the analysis of hand parameters, we effectively showcased the model’s interpretability and extended its applicability to videos recorded from different recording angles and distances by the strategic implementation of normalization and 3D keypoint rotation methods, addressing the initial concerns.

To validate accuracy and applicability, our Model-w-3D-tpi achieved high AAC rates of 88.0% for left-hand datasets and 81.5% for right-hand datasets (Supplementary Table 8). While the performance differs slightly between left and right hands due to separate training and testing datasets, our results demonstrate similar accuracy compared to previous studies^14,36,37^. We also assess consistency by calculating Kappa, which were 0.433 and 0.318 for left and right-hand FTTs, respectively. These coefficients align with the inter-rater consistency observed in Yang et al.’s research, with a range of Kappa from 0.28 to 0.66^4^. In the study by Williams et al.^38^ 22 neurologists rated the videos, and Kappa was only 0.28 ± 0.28. These findings suggest that our deep-learning model has reached a level of consistency comparable to human assessment.

Furthermore, our end-to-end model’s estimations are supported by clinical evidence-based hand parameters, such as frequency and intensity. We observed a negative correlation between frequency and intensity, both of which decreased as severity increased, consistent with clinical observations of bradykinesia. We also highlighted the use of 3D keypoint rotation in our estimation model to prevent overfitting and ensure accurate estimations across different recording angles.

From a clinical standpoint, Chen et al. previously demonstrated that quantitative hand parameters are related to clinical features^35^. Specifically, they found that calculated hand parameters obtained from tasks such as finger-tapping, open-closing, and pronation-supination can provide valuable insights into the patient’s disease status. Four notable clinical characteristics can be observed in the frequency and intensity evolution over time: hesitation and interruption (both frequency and intensity dip and recover during the task), amplitude decrement (the intensity decreases during the task), slowing (the frequency remains small over the whole time), and freezing or incapability to perform the task (the frequency is unchanged and small over time, and the patient cannot complete the task within a given time). Our analysis aligns with these findings and provides similar clinical feature insights through the utilization of time-series graphs. These graphs effectively portray the temporal dynamics of movement over a specified duration, allowing for comparisons with estimated scores to elucidate the predominant types of the motor impairment. While the FI value serves as a robust gauge of movement severity, it alone struggles to capture the intricacies of these four clinical characteristics. Presenting both frequency and intensity within a time-series plot, complemented by a keypoint-annotated video, offers an improved approach to aid clinicians in comprehending the individual’s real-time movement dynamics.

In terms of the pros and cons in the previous analysis frameworks for PD compared to our designs, previous commercialized AI-based evaluated systems, KELVIN^TM^ ^39^ and CloudUPDRS^40^, were developed to assist self-monitoring for PD. Machine Medicine Technologies Ltd. (London, UK) developed an all-in-one vision-based platform called KELVIN^TM^ (https://KELVIN.machinemedicine.com/) to assess PD patient movements with the MDS-UPDRS. This platform analyses hand movements and qualifies gait and rising from a chair in PD patients^14,17,41,42^. The computer-assessed scores with this platform have also been compared with clinical raters’ scores, and the platform showed acceptable consistency in multiple tasks^39^. In their recent research, they pooled over 10,000 videos from multiple sites and raters to improve model robustness^14^. However, limitations arise from using 2D keypoints extracted by OpenPose, such as the inability to perform 3D rotation and the requirement for the full human body in video analysis, making self-recording and immediate uploading challenging. On the other hand, the CloudUPDRS app was developed to address the challenge of home monitoring, using 16 smartphone-based tests to estimate subitems from the MDS-UPDRS part III, including rest tremor, hand postural tremor, finger tap, and leg agility. Jha et al. cross-validated the estimated score against blinded human raters by the app^43^, with the accuracy variably ranging from 53.2% to 97.0%. Although the CloudUPDRS provided several tests to assess each user’s motor functionality, there is no video of the movement that can be referenced as ground truth in the system. From the perspective of visualization, a video provides a user-friendly experience and more details than a curve of signals for clinicians. The physicians are also more familiar with the original tests in the MDS-UPDRS than with the tests used for the new system.

Compared with KELVIN^TM^ and CloudUPDRS, FastEval Parkinsonism provides a non-contactable video-based solution to estimate the severity of the motor movement remotely and instantly. We have demonstrated how our system deals with the issue of recording angles, thus providing multiple evaluation metrics, including MDS-UPDRS item score, frequency, intensity, intensity rate, and peak. Although we have not validated our findings in a large dataset, such as KELVIN^TM^, the outer validation by the PDMotorDB dataset alternatively confirms the validity with high AAC and the correlation between intensity and frequency. Unlike CloudUPDRS, our analysis is derived from the MDS-UPDRS test, making it easy for physicians to adapt and reduce their workload. The system allows independent viewing of files with raw video, evolving parameters, and evaluation metrics, enabling cross-validation and accurate diagnosis. Our radar plots compare the difference between left- and right-hand movements, aiding in the early detection of APD diseases. While KELVIN^TM^ and CloudUPDRS use multiple features, FastEval Parkinsonism focuses on finger taps but is ready to expand to additional parkinsonian features.

Regularly monitoring patients with our system and corresponding digital biomarkers allows us to vigilantly observe the possibility of clinical conversion from the diagnosis of PD to APD. Furthermore, our system holds the potential to contribute to the early detection of MPS or “mild motor signs” among the elderly population. This is of particular significance considering the elevated risk of neurodegenerative conditions in the elderly, extending beyond typical PD alone^29^. This approach lays the groundwork for the future development of personalized treatment plans.

While there are some limitations to acknowledge, we have opportunities for improvement. Firstly, enhancing accuracy remains a priority, and this can be achieved by expanding the size of our datasets. Fortunately, the collection of videos has become more efficient, thanks to the user-friendly system accessible through consumer-grade smartphones. Consequently, we are able to significantly enlarge our cohort dataset and improve our models by training them with a more extensive dataset. Second, our dataset was evaluated by only one clinician rater, which resolves the issue of the inter-rater discrepancy and benefits learning accurate mapping relationships among model-generated parameters, clinician-evaluated scores, and clinical observation. Nevertheless, the model might be more robust by including multiple raters at multiple sites in the future, as we found in the previous study^14^. Third, the demographics of the participants were older individuals who primarily spoke local languages, so the English version website was challenging for some users. To address this, a multi-language website should be developed to cater to different language preferences. Finally, we only cover one of the MDS-UPDRS tasks, finger taps. More parkinsonian features, such as hand open-close, hand protonation–supination, or gait performance, need to be automated and analyzed in the near future using a similar or updated platform as FastEval Parkinsonism. Despite the limitations, the adopted framework in our website provided a flexible and scalable to further new updates.

In conclusion, we developed FastEval Parkinsonism, a publicly accessible website that uses deep learning and quantitative calculation to analyze one of the parkinsonism movements, finger taps in patients with PD compared to participants with APD, healthy individuals, or elderly with MPS. Our findings showed that data augmentation techniques were useful in building the deep learning model and estimating scores for multi-angle videos. The optimized model accurately and effectively distinguished Parkinson’s symptom severity. FastEval Parkinsonism integrates analysis protocols into the Hand Predictor API, providing a valuable tool for self-assessment and assisting physicians in objectively monitoring the severity and symmetry of clinical symptoms over time.

## Methods

### Study design and data collection

Patients and healthy subjects participating in this study were recruited from two hospitals, NTUH and NTUCC, during the period from October 19, 2020, to August 31, 2022. The patients with PD, MSA, mild cognitive impairment (MCI), Alzheimer’s disease (AD)^44^, and HC were diagnosed and classified by Dr. Ming-Che Kuo. The patients with MSA were categorized as the participants with APD, while those with MCI and AD were classified as the elderly with mild parkinsonian signs (MPS)^28,29^. HCs were defined as those with no neurogenerative disease but with the same age and sex distribution as the other patient groups. All subjects provided consent for data management and usability prior to data collection. Each subject performed a FTT twice for each hand, which was recorded by a ZED camera with 720p (width: 720 pixels, height: 1280 pixels) and 60 frames per second (fps) in a side-view. Every person continuously performed finger taps for approximately 10 s in this study; this approach differs slightly from that used for the MDS-UPDRS. The MDS-UPDRS item score for the FTT was evaluated by an experienced movement disorder specialist, considering speed, amplitude, and parkinsonism features. Each video received an independent score from 0 to 4. The scores were then transformed into multiple binary subtasks for simplification. Due to limited data, MDS-UPDRS item score 3 and 4 were combined into a single group (score of 3+) in this study.

#### Ethics approval

All subjects provided written informed consent, and the study was approved by Research Ethics Committees at NTUH (201809022RINA and 202108149RINA).

### Dataset splitting and cross-validation

The dataset was split based on patients to avoid bias. The non-testing and testing datasets were uniformly randomly divided with a ratio of 0.85 to 0.15 using a Python package, Random, resulting in 362 video clips in the former and 58 clips in the latter dataset for each hand side. The non-testing dataset was further divided into training and validation datasets. Hyperparameter optimization involved 3-fold cross-validation for classifier performance assessment and 5-fold cross-validation for training binary classifiers. A lower cross-validation fold was used for performance assessment to expedite the process of grid-searching hyperparameters. However, once a set of optimal hyperparameters was identified, we switched to a higher cross-validation fold to train models with a more extensive dataset, thereby expanding the feature spaces. The classifier with the highest validation MCC was chosen for MDS-UPDRS item score estimation.

### Data processing

There are various tools for human keypoint detection, including OpenPose^13^, Detectron2^45^, MMPose^46^, AlphaPose^47^, and MediaPipe^27^. MediaPipe was chosen for this study due to its superior performance in 3D hand keypoint estimation^48^. The MediaPipe Hands API^49^ was used with specific configurations to extract 21 3D hand keypoints from each video frame.

To address potential misunderstandings and malfunctions regarding the extracted 3D keypoints, we introduced the “error frames ratio (EFR)” as a measure of video quality. This concept is inspired by the bit error rate and frame error rate (FER) used in data processing for communication systems^50,51^. The EFR is defined as the proportion of frames in which hand keypoints were not successfully extracted, relative to the total number of frames in the video. Three categories were established to indicate video confidence: high (EFR < 0.16), moderate (EFR between 0.16 and 0.5), and low (EFR ≥ 0.5).

Keypoints with low confidence were discarded, and each video was assumed to contain one person or one hand. Raw keypoints were normalized using the length of the thumb as a factor. Thumb length was determined by summing the Euclidean distances between the four thumb keypoints and one palm keypoint. The missing values would be supplied by tracking back to find the nearest valid hand keypoints to fill in the missing values and make hand parameter analysis more reasonable. The processing method reduced the programming jitter and promoted the continuity of data.

### Deep learning modeling

The MDS-UPDRS item score estimation was a multi-label (score 0, 1, 2, 3+) classification task, whereas some classes had less than enough data to train well in an end-to-end model. Thus, this study used a binary estimation approach by classifiers with different boundaries, which improved the overall accuracy of the estimated MDS-UPDRS item score by combining the estimations from different classifiers with different boundaries, providing a more robust and unbiased final score.

Three neural network architectures were compared to find a suitable and efficient to reach the goal of instant score estimation. First, we considered the original PDHandNet proposed by Ho^31^, which is lightweight and demonstrates good performance, making it well-suited for our web platform that aims to provide rapid results. Second, we evaluated a modified PDHandNet, enhanced with an additional dilated convolutional block, linear layer, and ReLU layer (see Supplementary Fig. 7), to determine if a slightly deeper version could offer improved performance. Lastly, we examined the multichannel convolutional neural network with gated recurrent units (CNN-GRU) model, proposed by Lu et al.^32^ known for its outstanding performance in classification tasks involving time-series data. We did not consider the traditional LSTM model due to its inferior performance, nor did we consider the prevalent transformer-based architecture because of its complexity and the difficulty in training it with our limited dataset.

Among these three architectures, we initially filtered out the most efficient model architecture suitable for our score classification subtasks, using the left-hand finger-tapping dataset. The MCC metric was chosen for this primary performance comparison, as it is more effective than accuracy and the F1 score in evaluating the performance of a binary task^52^.

Subsequently, we focused on hyperparameter grid-searching to identify an optimal model for the binary subtask with item scores of 0 and 1+ (scores: 1, 2, 3+). We conducted searches with batch sizes of 16 and 64, learning rates of 1e-3 and 1e-4, and L2 regularization values of 5e-4, 5e-5, and 5e-6. All training was optimized using stochastic gradient descent (SGD) with a momentum of 0.9 and a cross-entropy loss function.

#### Data augmentation

Data augmentation could enhance the robustness of the models and make models more generalized. It was applied at three stages: training, model-picking, and inference. Adding data augmentation at the training stage could be regarded as expanding the training space and giving more features for building a generalized model, reducing overfitting, and then improving the validation performance^53,54^.

Two data augmentation techniques, 3D keypoint rotation and random video cropping, derived from a previous study^31^, were employed to expand the training dataset. First, we implemented 3D rotation for hand keypoints since target users of this system might upload their videos from different angles. Second, since the designed model only accepted the fixed length of the data, the raw training data should be cropped before being used to model training. Random video cropping was used not only to solve the issue of the input data length but also to enlarge the dataset for model training. Gaussian random sampling was proposed to extract the middle clips of the whole videos and reduce recording error without manual trimming.

Random cropping augmentation applied during inference can further improve model performance, akin to ensembling. This method allows the model to make predictions on different data segments, which are then aggregated to produce the final output.

### Evaluation metrices

For binary classification tasks, several indices were included in this study to evaluate the model performance. The definition of the evaluation metrics for binary classification tasks are shown in Eqs. (1) to (6). Compared to the F1 score, the MCC is regarded as a more informative and truthful score for a binary task^52^.

1 $${\mathrm{Accuracy}}=\frac{{\mathrm{TP}}+{\mathrm{TN}}}{{\mathrm{TP}}+{\mathrm{FP}}+{\mathrm{FN}}+{\mathrm{TN}}}$$

2 $${\mathrm{Sensitivity}}\,({\mathrm{Recall}})=\frac{{\mathrm{TP}}}{{\mathrm{TP}}+{\mathrm{FN}}}$$

3 $${\mathrm{Specificity}}=\frac{{\mathrm{TN}}}{{\mathrm{TN}}+{\mathrm{FP}}}$$

4 $${\mathrm{Precision}}=\frac{{\mathrm{TP}}}{{\mathrm{TP}}+{\mathrm{FP}}}$$

5 $${\rm{F}}1{\rm{score}}={{2}}\times \frac{{\mathrm{Sensitivity}}\times {\mathrm{Precision}}}{{\mathrm{Sensitivity}}+{\mathrm{Precision}}}$$

6 $${\mathrm{Matthews}}\,{\mathrm{correlation}}\,{\mathrm{coefficient}}\,({\mathrm{MCC}})=\frac{{\mathrm{TP}}\times {\mathrm{TN}}-{\mathrm{FP}}\times {\mathrm{FN}}}{\sqrt{({\mathrm{TP}}+{\mathrm{FP}})({\mathrm{TP}}+{\mathrm{FN}})({\mathrm{TN}}+{\mathrm{FP}})({\mathrm{TN}}+{\mathrm{FN}})}}$$

where **TP** is true positive, **TN** is true negative, **FP** is false positive, and **FN** is false negative.

Three evaluation metrics were used in this study for multiple-label classification—the number of significant error files (NSE), acceptable accuracy (AAC)^36^, and Cohen’s kappa coefficient^55^. These are detailed in Eqs. (7) to (9).

7 $${\mathrm{The}}\,{\mathrm{number}}\,{\mathrm{of}}\,{\mathrm{significant}}\,{\mathrm{error}}\,{\mathrm{files}}({\mathrm{NSE}})={{\mathrm{N}}}_{{\rm{|}}{\mathrm{y}}-\hat{{\mathrm{y}}}{\rm{|}}\ge {\mathrm{2}}}$$

8 $${\mathrm{Acceptable}}\,{\mathrm{accuracy}}\,({\mathrm{AAC}})=\frac{{{\mathrm{N}}}_{{\rm{|}}{\mathrm{y}}-\hat{{\mathrm{y}}}{\rm{|}}\le {\mathrm{1}}}}{{\mathrm{N}}}$$

9 $$\begin{array}{l}{\mathrm{Cohen}}{\rm{\mbox{'}}}{\mathrm{s}}\,{\mathrm{kappa}}\,{\mathrm{coefficient}}\,({\mathrm{Kappa}})= \displaystyle\frac{{{\mathrm{p}}}_{{\mathrm{o}}}-{{\mathrm{p}}}_{{\mathrm{e}}}}{{\mathrm{1}}-{{\mathrm{p}}}_{{\mathrm{e}}}}\\\quad{\mathrm{where}}\,{{\mathrm{p}}}_{{\mathrm{o}}}=\displaystyle\frac{{{\mathrm{N}}}_{\left|{\mathrm{y}}-\hat{{\mathrm{y}}}\right|={\mathrm{0}}}}{{\mathrm{N}}},{{\mathrm{p}}}_{{\mathrm{e}}}=\displaystyle\frac{{\mathrm{1}}}{{{\mathrm{N}}}^{{\mathrm{2}}}}\mathop{\sum}\limits _{{\mathrm{k}}}{{\mathrm{n}}}_{{\mathrm{k}},{\mathrm{y}}}{{\mathrm{n}}}_{{\mathrm{k}},\hat{{\mathrm{y}}}}\end{array}$$

where **N** is the number of observations, **y** is the label, $\hat{{\bf{y}}}$ is the estimated result, and **n**_**k,i**_ is the number of times rater **i** estimated category **k**.

### Hand parameters

For the FTT, the representative parameter is the Euclidean distance between the tips of the thumb and index finger. This study used a 5-frame moving average filter on the hand parameter to reduce the jitter error from the keypoint extractor, MediaPipe. The keypoints were initially standardized based on each patient’s thumb-length to optimize for hand size variations within video clips, which could arise from diverse real-world recording settings. The peak of the hand parameter was extracted with a prominence of 0.1 (10% of the thumb’s length of the subject), whereas frequency and intensity were extracted by the STFT. Furthermore, a more comprehensive metric, FI value, was calculated by finding the dot product of frequency and intensity in each time step, representing their transient combined motor performance.

### Website design

We combined MDS-UPDRS estimation and hand parameter calculation in an API referred to as Hand Predictor to obtain results from the raw video. The statistics of each hand parameter were calculated to represent the performance of the hand movement, which was displayed on the FastEval Parkinsonism webpage. The FastEval Parkinsonism website was built using Ruby on Rails (version 7.0.4) with a model-view-controller framework, following the principles of Don’t Repeat Yourself and Convention Over Configuration. The account management system utilized the devise package (version 4.8.1) for efficient development. Additionally, a queuing system was implemented using Sidekiq (version 7.0.2) and Redis (version 7.0.7) to handle data processing and resource allocation efficiently for multiple users.

### Reporting summary

Further information on research design is available in the Nature Research Reporting Summary linked to this article.

### Supplementary information

Supplementary

Reporting Summary

## Data Availability

Due to the patient data privacy policy, the original dataset (video clips) would not be publicly accessed. The de-identified data that support the findings of this study are available from the corresponding author upon reasonable request, with the permission of the institution, and after approval of a proposal.

## Code availability

Custom codes were implemented in Python 3.8.12 with PyTorch 1.11.0 and run on an Ubuntu 18.04 or 22.04 system with NVIDIA GTX 1080 Ti and RTX 3060 Ti. Source codes are available at a GitHub repository (https://github.com/yuyuan871111/fast_eval_Parkinsonism) under an Apache-2.0 license.
