# Supplementary material for: FastEval Parkinsonism: an instant deep learning–assisted video-based online system for Parkinsonian motor symptom evaluation
Source: NPJ Digit Med. 2024 Feb 8;7:31. doi: 10.1038/s41746-024-01022-x (PMC10853559; doi:10.1038/s41746-024-01022-x)
Supplement: Supplementary file 1 — Supplementary [file 41746_2024_1022_MOESM1_ESM.pdf]

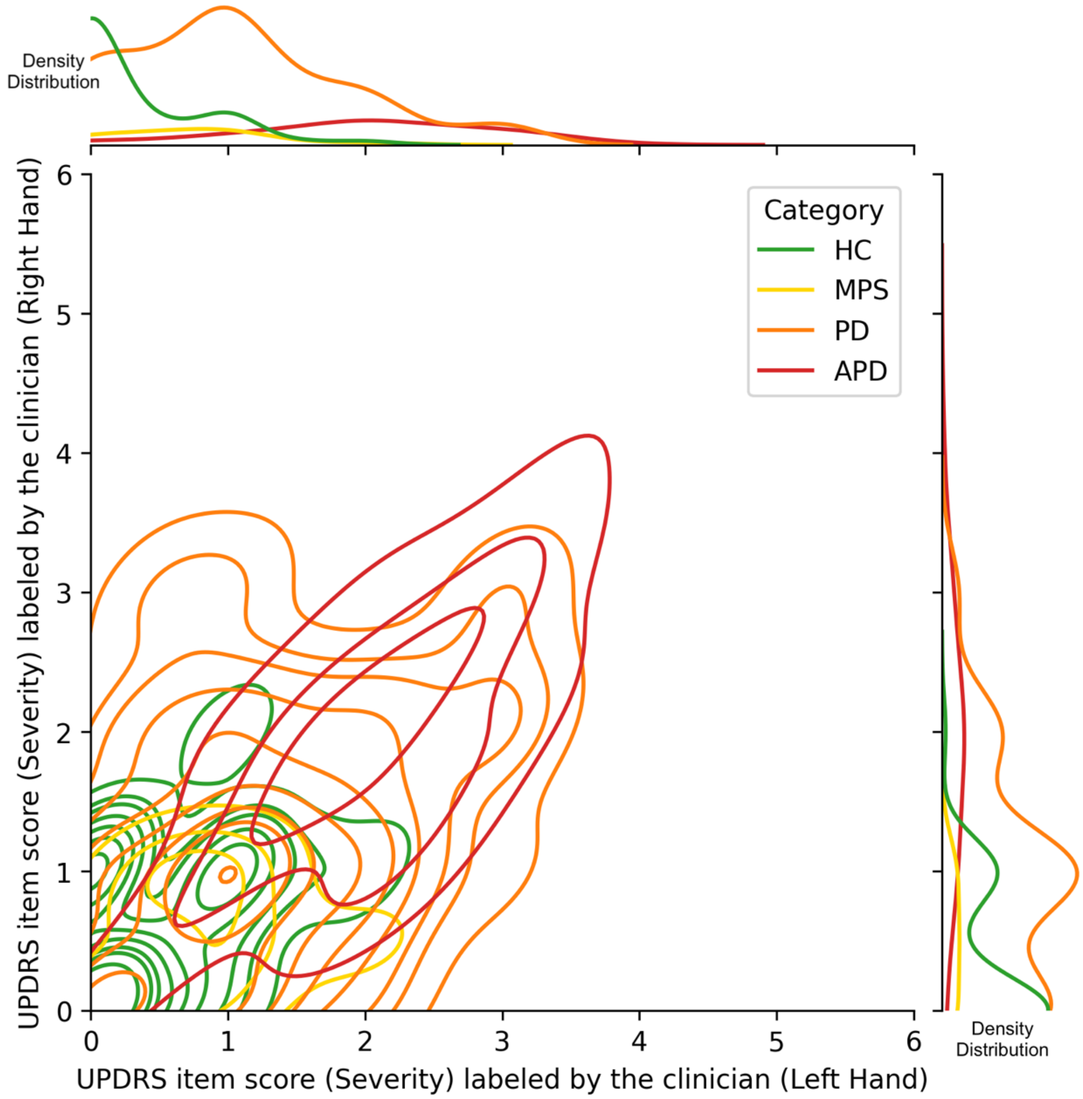

Supplementary Figure 1. The distribution of the UPDRS item score for both hands in each patient's visit in four groups.

During each patient's visit, two clips were recorded for each hand. These clips were independently scored, and the average of these two scores was taken as the final score for that particular hand. A total of 210 visits were included in this analysis. The distribution of the scores in each group were represented using bivariate and univariate kernel density estimations (KDEs). HC: healthy controls; MPS: the elderly with mild parkinsonism signs; PD: patients with Parkinson's disease; APD: patients with atypical parkinsonism.

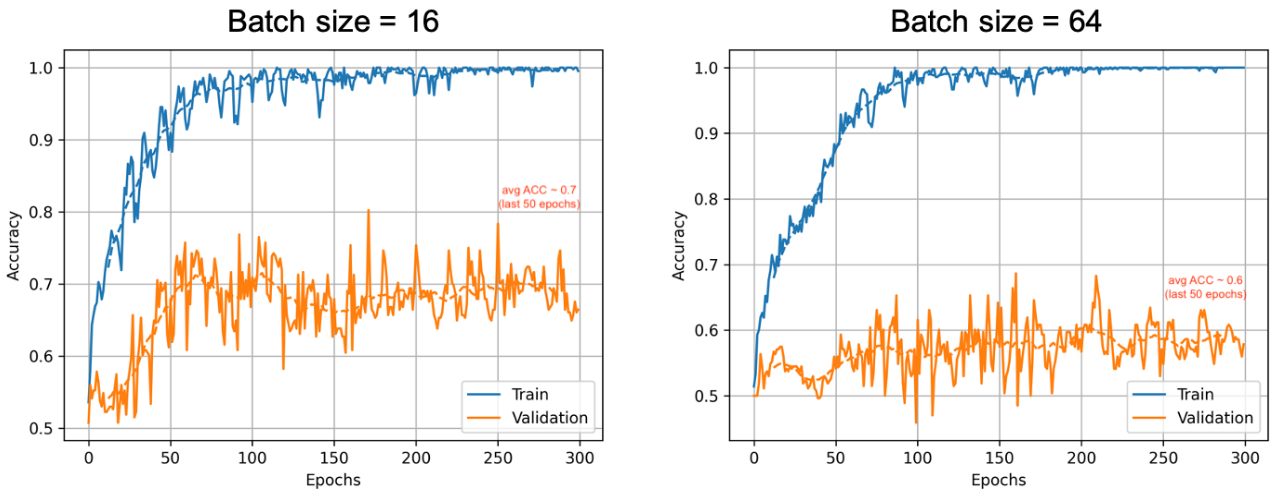

Supplementary Figure 2. Comparing model training accuracy with different batch sizes over 300 epochs on training and validation datasets.

To assess the effect of different batch sizes on model performance, we maintained fixed hyperparameters (learning rate: 0.001, L2 regularization: 0.00005, threshold of error frames ratio: 0.5, times of oversampling: 2, all 21 keypoints included). To enhance model accuracy and escape local minimums, we applied a cosine annealing scheduler with a period of 10 epochs. Surprisingly, while larger batch sizes improved training smoothness, using smaller batch sizes resulted in a remarkable 10% increase in average model accuracy on the validation dataset compared to larger batch sizes. This suggests the successful escape from some local minimums during training. The dashed line represents the moving average of the accuracy.

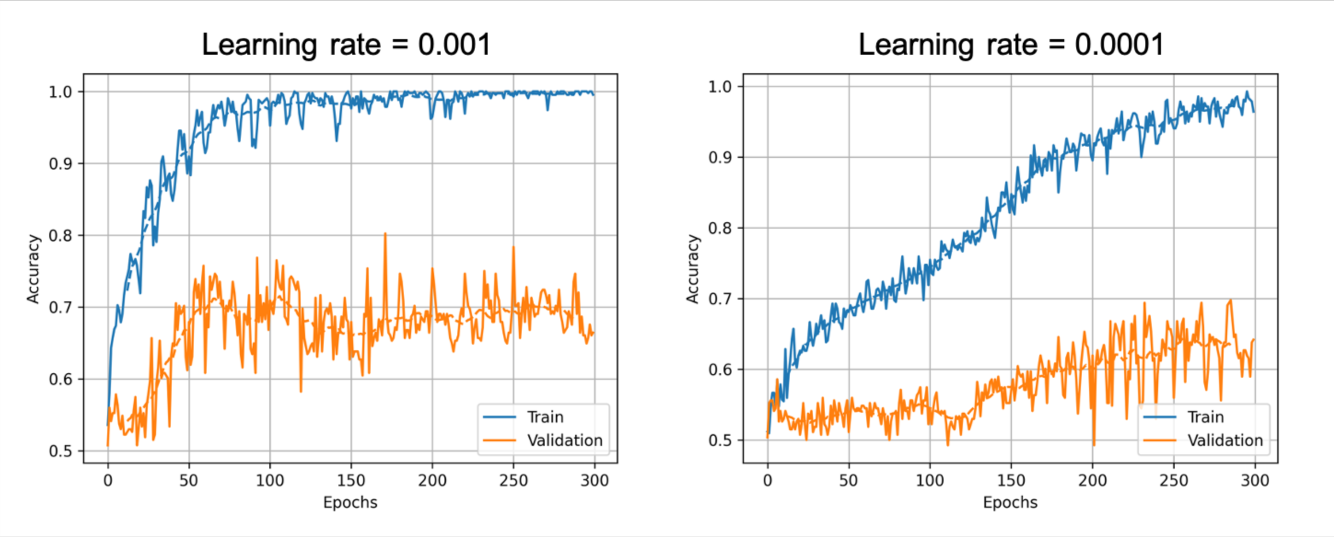

Supplementary Figure 3. Comparing model training accuracy with different learning rates over 300 epochs on training and validation datasets.

To assess the effect of different learning rates on model performance, we fixed other hyperparameters (batch size: 16, L2 regularization: 0.00005, threshold of error frames ratio: 0.5, times of oversampling: 2, all 21 keypoints included). To improve model accuracy and circumvent local minimums, a cosine annealing scheduler with a 10-epoch period was used. Notably, a larger learning rate accelerates convergence, albeit with relatively higher fluctuations. In terms of selecting an optimal model, training with a larger learning rate occasionally resulted in higher accuracy during the training process, allowing us to preserve those superior models for subsequent applications. The dashed line represents the moving average of the accuracy.

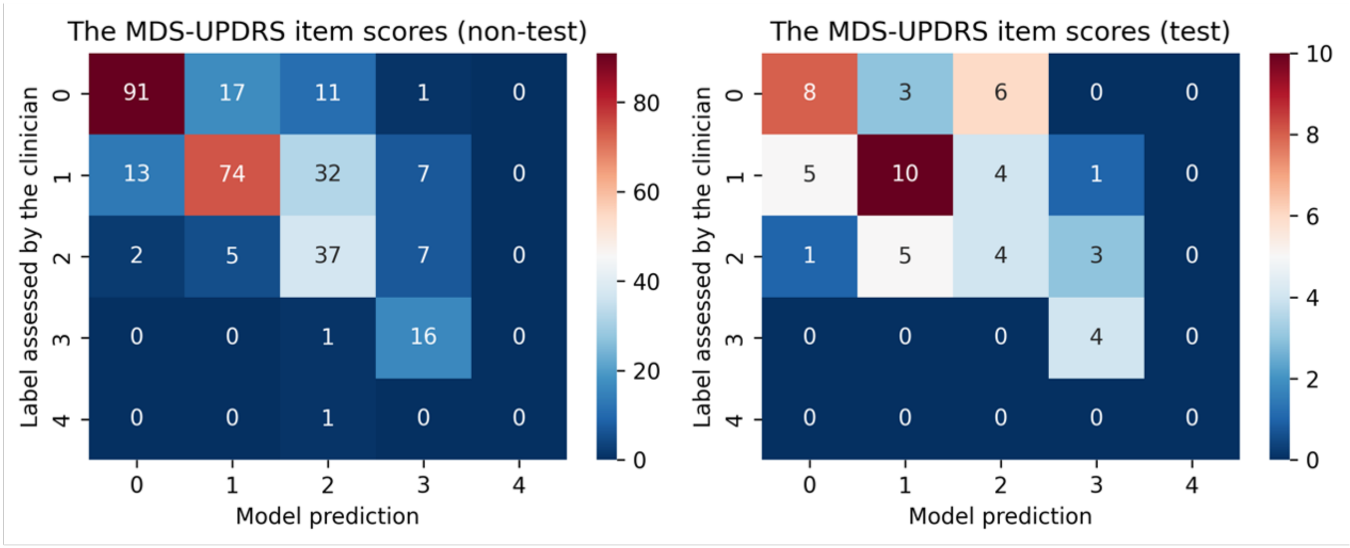

Supplementary Figure 4. Confusion matrix of the MDS-UPDRS item scores assessed by the clinician and the best-selected model (Model-w-3D-ti) for the right-hand finger tapping task in non-testing and testing dataset.

The number in the confusion matrix represents the number of files.

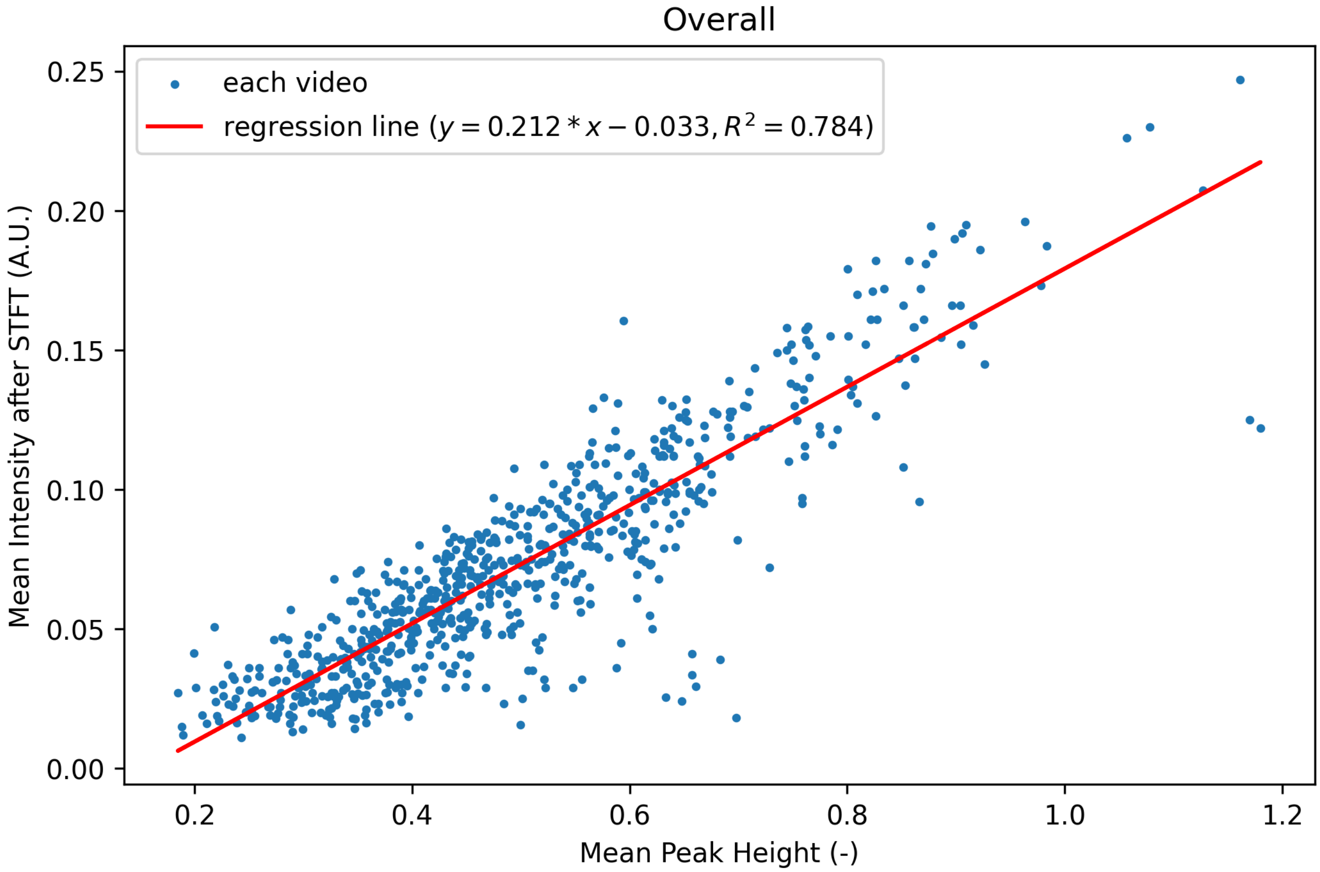

Supplementary Figure 5. The overall relationship between the peak and intensity in our dataset. The dataset comprises video clips capturing both left- and right-hand movements. For each video, the mean peak height and intensity of the oscillatory hand movement (e.g. finger taps) were computed. To establish the relationship between these two parameters, a linear regression analysis was conducted, resulting in an R^2 value of 0.784. This coefficient indicates a strong correlation between the peak height and intensity, suggesting a significant association between these variables. STFT: short-time Fourier transform.

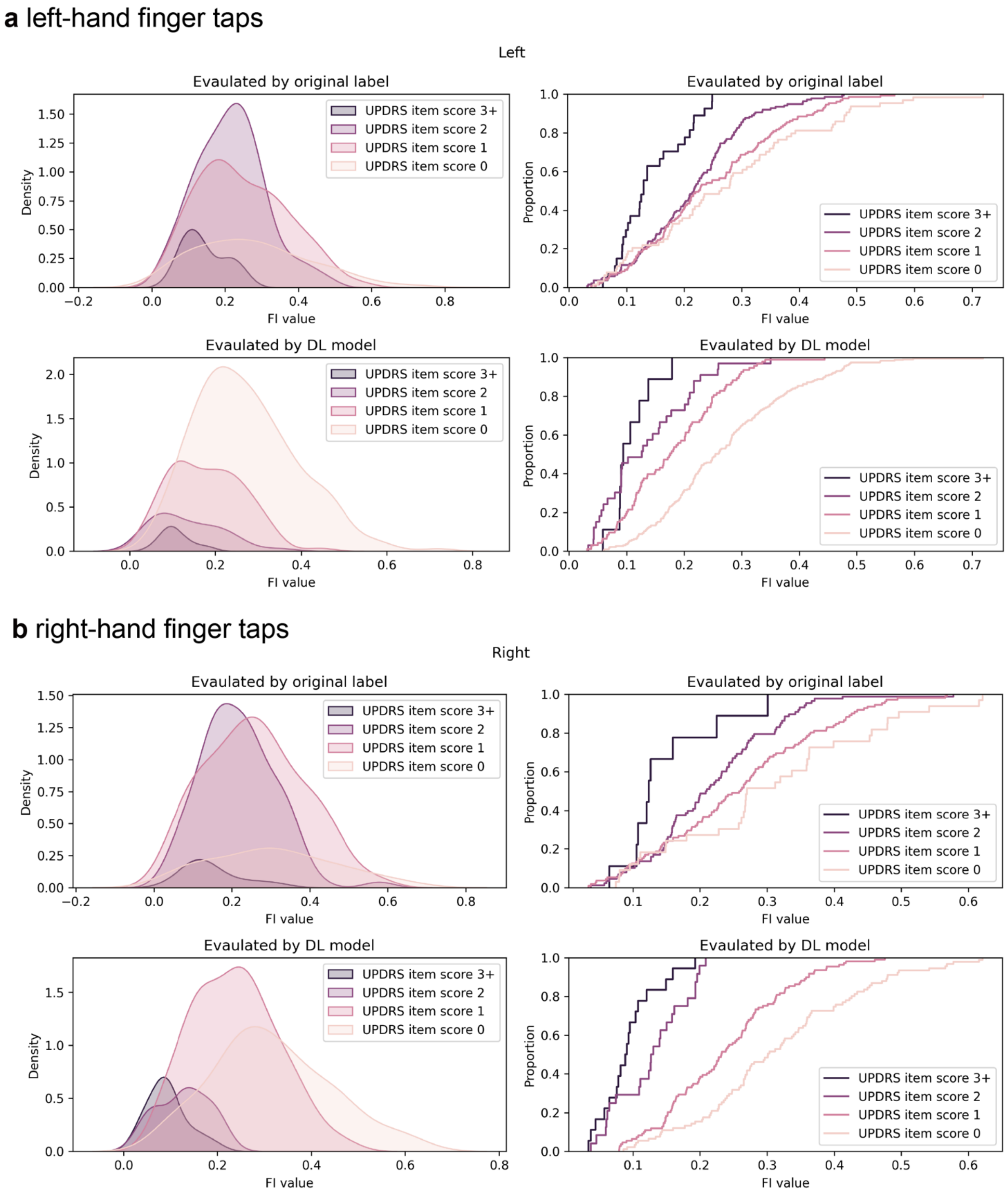

Supplementary Figure 6. Distribution and cumulative percentage of the averaged FI value in each MDS-UPDRS item scored by original evaluators and Model-w-3D-tpi in the PDMotorDB dataset. Similar to Figure 4, the distribution of the FI value in each score group illustrates the connection between model-predicted scores and a representative hand parameter, the FI value. The cumulative percentage of

the averaged FI values highlights discernible differences and boundaries among the different score groups. Notably, the FI value signifies the speed of motor movement, making higher values more desirable for healthy individuals.

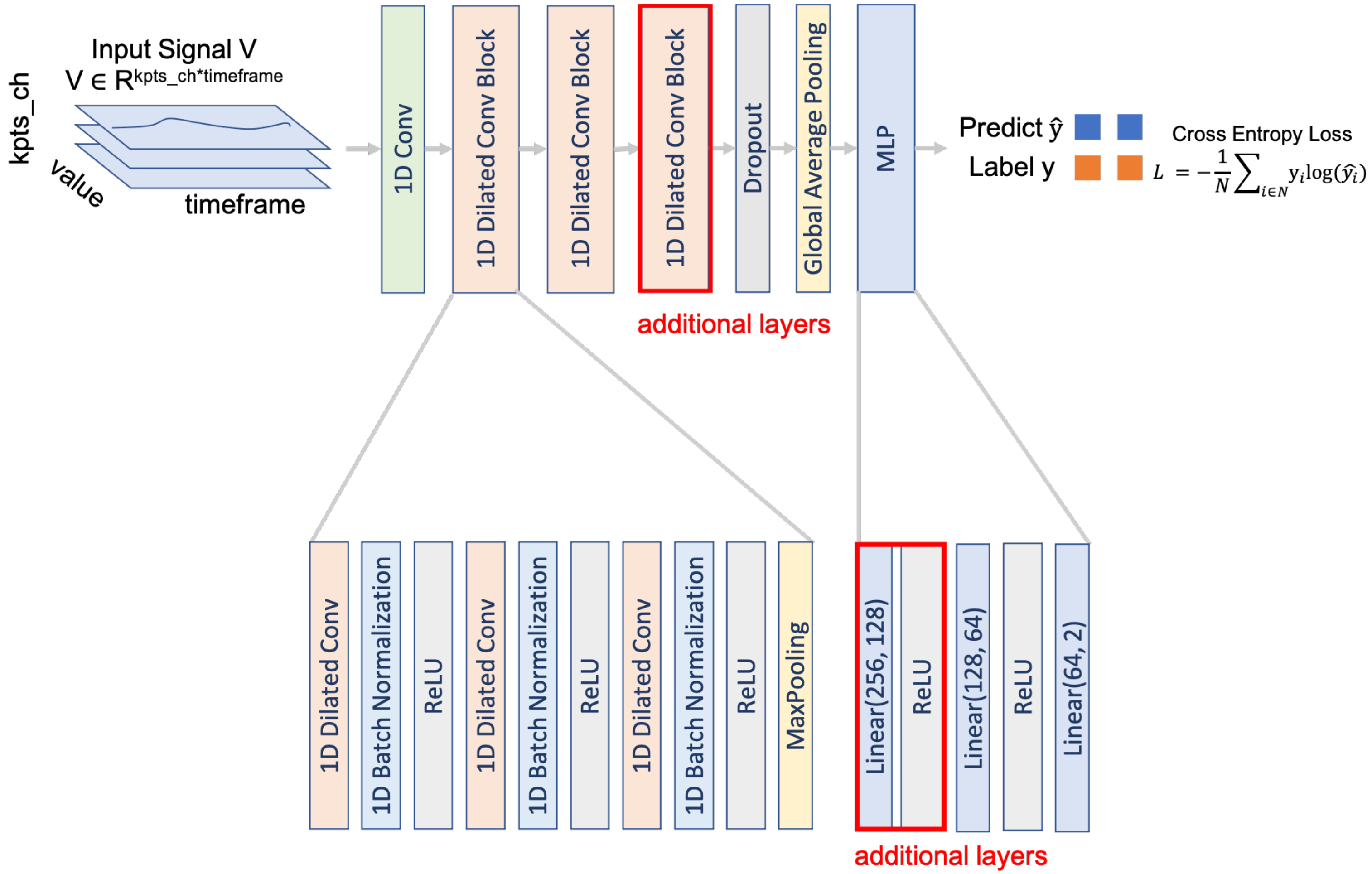

Supplementary Figure 7. The architecture of the modified PDHandNet (deeper version).

1D Conv: 1D convolutional layer; 1D Dilated Conv: 1D dilated convolutional layer; MLP: multiple layer perceptron. The 1D dilated convolutional block before the dropout block and the linear layer (Linear (256, 128)) with a ReLU layer in the MLP block were added to the modified PDHandNet compared to the original one.

Supplementary Table 1. The statistics of participants and visits in four groups.

For the age and gender, the statistics was calculated based on the participants; for the UPDRS item scores and the symmetry of hand movement impairment, similar to Supplementary Figure 1, two scores for each hand were averaged based on each visit. Subsequently, the overall scores were obtained by averaging the scores from both the right and left hands. In each group, the median, mean, and standard deviation (STD) were calculated. Within each group, the difference between the right-hand score and the left-hand score was calculated, and the absolute value of this difference (AVD) was categorized and tallied according to severity. HC: Healthy controls; MPS: the elderly with mild parkinsonism signs; PD: patients with Parkinson's disease; APD: patients with atypical parkinsonism.

**Group**				**HC**	**MPS**	**PD**	**APD**
**The statistics of participants**							
The number of participants				47	12	103	24
Ages – mean ± STD (years old)				65.4 ± 12.9	74.8 ± 6.0	70.8 ± 8.2	59.5 ± 8.1
Ages – median (years old)				67.0	74.5	71.0	62.0
Male (%)				22 (46.8%)	5 (41.7%)	58 (56.3%)	17 (70.8%)
**The UPDRS item score for both hands in each patient's visit**							
The number of participants' visits				48	12	123	27
Overall – mean ± STD				0.3 ± 0.4	0.6 ± 0.4	1 ± 0.7	1.9 ± 0.9
Overall – median				0.1	0.8	1	2
Right hand – mean ± STD				0.4 ± 0.5	0.5 ± 0.4	1 ± 0.8	1.9 ± 0.9
Right hand – median				0	0.5	1	2
Left hand – mean ± STD				0.3 ± 0.5	0.7 ± 0.6	1 ± 0.8	2 ± 0.9
Left hand – median				0	0.8	1	2
**The symmetrical and asymmetrical hand movement impairment in each patient's visit**							
**Type**	**Dominant**	**AVD**	**Severity**	-	-	-	-
Asymmetry	Left	> 2	Severe	0 (0%)	0 (0%)	0 (0%)	0 (0%)
		(1, 2]	Mild	0 (0%)	1 (8%)	8 (7%)	1 (4%)
Undefined (buffer zone)		(0, 1]	Slight	8 (17%)	4 (33%)	40 (32%)	7 (26%)
Symmetry	-	0	-	30 (62%)	4 (33%)	43 (35%)	13 (48%)
Undefined (buffer zone)	Right	(0, 1]	Slight	10 (21%)	3 (26%)	23 (18%)	6 (22%)
Asymmetry		(1, 2]	Mild	0 (0%)	0 (0%)	7 (6%)	0 (0%)
		> 2	Severe	0 (0%)	1 (8%)	2 (2%)	0 (0%)
The ratio of asymmetry to symmetry				0	0.25	0.40	0.077

Supplementary Table 2. The confusion matrix of the MDS-UPDRS evaluation for the left-hand finger-tapping task by a clinician and a trained evaluator.

Clinician \ Trained evaluator	0	1	2	3	4	Total
**0**	125	30	0	0	0	155
**1**	20	107	30	0	0	157
**2**	0	20	46	9	0	75
**3**	0	0	12	20	0	32
**4**	0	0	0	1	0	1
**Total**	145	157	88	30	0	420

Supplementary Table 3. The confusion matrix of the MDS-UPDRS evaluation for the right-hand finger-tapping task by a clinician and a trained evaluator.

Clinician \ Trained evaluator	0	1	2	3	4	Total
**0**	128	36	0	0	0	164
**1**	17	116	23	0	0	156
**2**	0	17	52	5	0	74
**3**	0	0	3	21	0	24
**4**	0	0	0	2	0	2
**Total**	145	169	78	28	0	420

Supplementary Table 4. The consistency of the MDS-UPDRS evaluation of the finger tapping between a clinician and a trained evaluator.

The side of the hands	The number of significant error files (NSE)	Acceptable accuracy (AAC)	Cohen's kappa coefficient	Level of agreement	The proportion of the reliable data
Left	0	1 (100%)	0.5790	Weak	15% ~ 35%
Right	0	1 (100%)	0.6389	Moderate	35% ~ 63%

Supplementary Table 5. Data quality effect on model performance for the score 0/1+ binary classification subtask (finger tapping, non-testing dataset).

Based on varying thresholds of error frames ratio (TEFR), clips with a high error frames ratio were carefully filtered. While more training data can enrich the model's features, it is essential to be cautious about the quality of the clips, as low-quality ones may introduce noise to the models. A two-tailed Student's t-test ($\alpha = 0.05$) was conducted, revealing only marginal difference between models with varying TEFR (p-value > 0.05). A set of optimized hyperparameters were chosen (batch size: 16, learning rate: 0.001, L2 regularization: 0.00005, times of oversampling: 2, all 21 keypoints included). 3-fold cross-validation was applied for model performance assessment. Acc: accuracy; Sens: sensitivity; Spec: specificity; Prec: precision; F1: F1 score; MCC: Matthews correlation coefficient.

**TEFR**	**Number of video clips passing the TEFR**	**Dataset**	**Acc**	**Sens**	**Spec**	**Prec**	**F1**	**MCC**
**Left hand**								
0	88	-	-	-	-	-	-	-
0.3	272	Training	0.96±0.03	0.96±0.04	0.97±0.03	0.97±0.03	0.96±0.03	0.93±0.07
		Validation	0.74±0.03	0.78±0.08	0.69±0.02	0.72±0.01	0.75±0.04	0.48±0.07
0.5	308	Training	0.96±0.02	0.97±0.03	0.95±0.01	0.96±0.02	0.96±0.02	0.92±0.04
		Validation	0.78±0.06	0.82±0.04	0.73±0.15	0.77±0.09	0.79±0.04	0.56±0.11
1	362	-	-	-	-	-	-	-
**Right hand**								
0	0	-	-	-	-	-	-	-
0.3	280	Training	0.97±0.02	0.97±0.03	0.98±0.02	0.98±0.02	0.97±0.03	0.95±0.05
		Validation	0.75±0.03	0.79±0.08	0.71±0.11	0.74±0.05	0.76±0.02	0.51±0.05
0.5	315	Training	0.99±0.01	0.99±0.01	0.99±0.01	0.99±0.01	0.99±0.01	0.98±0.01
		Validation	0.76±0.02	0.83±0.07	0.70±0.09	0.74±0.05	0.78±0.01	0.54±0.02
1	362	-	-	-	-	-	-	-

Supplementary Table 6. The performance of the models with different neural network architectures for the left-hand finger-tapping binary classification subtask to distinguish the score of 0 and 1+. Acc: accuracy; Sens: sensitivity; Spec: specificity; Prec: precision; F1: F1 score; MCC: Matthews correlation coefficient.

**Model name**	**Acc**	**Sens**	**Spec**	**Prec**	**F1**	**MCC**
Original PDHandNet	Training dataset					
	0.83±0.04	0.84±0.03	0.81±0.06	0.82±0.05	0.83±0.04	0.65±0.08
	Validation dataset					
	0.69±0.04	0.71±0.04	0.67±0.11	0.69±0.07	0.69±0.03	0.38±0.08
Modified PDHandNet (More layers)	Training dataset					
	0.83±0.04	0.83±0.04	0.83±0.05	0.83±0.05	0.83±0.04	0.66±0.09
	Validation dataset					
	0.64±0.06	0.77±0.09	0.51±0.13	0.62±0.06	0.68±0.05	0.30±0.12
Multichannel CNN-GRU	Training dataset					
	0.81±0.05	0.83±0.03	0.79±0.07	0.80±0.05	0.81±0.04	0.61±0.09
	Validation dataset					
	0.68±0.06	0.65±0.07	0.72±0.14	0.71±0.10	0.67±0.06	0.38±0.13

Supplementary Table 7. The best performance of the models with different 3D keypoint rotation settings for the left-hand finger-tapping 0/1+ binary classification subtask.

The best model was saved in a certain epoch during model training, also listed in the table. The 5-fold validation policy separated the training and validation dataset. The models annotated with bold texts show better performance in testing dataset as 3D keypoint rotation is implemented at the inference stage. Model-wo-3D: basic model without 3D rotation; Model-w-3D-t: model with 3D rotation at the training stage only; Model-w-3D-ti: model with 3D rotation at training and inference stage; Model-w-3D-tp: model with 3D rotation at training and model-picking stage; Model-w-3D-tpi: model with 3D rotation at training, model-picking and inference stage; Acc: accuracy; Sens: sensitivity; Spec: specificity; Prec: precision; F1: F1 score; MCC: Matthews correlation coefficient.

**Model name**	**Epoch (save at)**	**Dataset**	**Acc**	**Sens**	**Spec**	**Prec**	**F1**	**MCC**
Model-wo-3D	121	Training	0.97	0.96	0.98	0.98	0.97	0.94
		Validation	0.77	0.70	0.83	0.81	0.75	0.54
		Testing	0.71	0.64	0.87	0.91	0.75	0.47
Model-w-3D-t	278	Training	0.84	0.84	0.84	0.84	0.84	0.67
		Validation	0.83	0.78	0.88	0.87	0.82	0.66
		Testing	0.73	0.79	0.60	0.81	0.80	0.38
**Model-w-3D-ti**	**278**	**Training**	**0.84**	**0.84**	**0.84**	**0.84**	**0.84**	**0.67**
		**Validation**	**0.83**	**0.78**	**0.88**	**0.87**	**0.82**	**0.66**
		**Testing**	**0.79**	**0.79**	**0.80**	**0.90**	**0.84**	**0.56**
Model-w-3D-tp	258	Training	0.81	0.81	0.82	0.82	0.81	0.62
		Validation	0.80	0.76	0.84	0.83	0.79	0.60
		Testing	0.67	0.70	0.60	0.79	0.74	0.28
**Model-w-3D-tpi**	**258**	**Training**	**0.81**	**0.81**	**0.82**	**0.82**	**0.81**	**0.62**
		**Validation**	**0.80**	**0.76**	**0.84**	**0.83**	**0.79**	**0.60**
		**Testing**	**0.77**	**0.79**	**0.73**	**0.87**	**0.83**	**0.50**

Supplementary Table 8. The performance of the models with different 3D keypoint rotation settings for predicting the MDS-UPDRS item scores for finger taps.

Bold text represents the best among the settings. Model-wo-3D: basic model without 3D rotation; Model-w-3D-t: model with 3D rotation at the training stage only; Model-w-3D-ti: model with 3D rotation at training and inference stage; Model-w-3D-tp: model with 3D rotation at training and model-picking stage; Model-w-3D-tpi: model with 3D rotation at training, model-picking and inference stage; NSE: the number of significant error files; AAC: acceptable accuracy; Kappa: Cohen's kappa coefficient.

**Model name**	**Dataset**	**NSE**	**AAC**	**Kappa**
**Left hand**				
Model-wo-3D	Overall	9	0.975 (97.5%)	0.688
	Non-testing	4	0.987 (98.7%)	0.742
	Testing	5	0.900 (90.0%)	0.362
Model-w-3D-t	Overall	62	0.827 (82.7%)	0.376
	Non-testing	54	0.825 (82.5%)	0.392
	Testing	8	0.840 (84.0%)	0.275
Model-w-3D-ti	Overall	29	0.919 (91.9%)	0.535
	Non-testing	23	0.925 (92.5%)	0.556
	Testing	6	0.880 (88.0%)	0.407
Model-w-3D-tp	Overall	33	0.908 (90.8%)	0.353
	Non-testing	26	0.916 (91.6%)	0.382
	Testing	7	0.860 (86.0%)	0.178
**Model-w-3D-tpi**	**Overall**	**19**	**0.947 (94.7%)**	**0.492**
	**Non-testing**	**13**	**0.958 (95.8%)**	**0.501**
	**Testing**	**6**	**0.880 (88.0%)**	**0.433**
**Right hand**				
Model-wo-3D	Overall	14	0.962 (96.2%)	0.750
	Non-testing	4	0.987 (98.7%)	0.840
	Testing	10	0.815 (81.5%)	0.261
Model-w-3D-t	Overall	33	0.911 (91.1%)	0.359
	Non-testing	23	0.927 (92.7%)	0.386
	Testing	10	0.815 (81.5%)	0.202
**Model-w-3D-ti**	**Overall**	**30**	**0.919 (91.9%)**	**0.520**
	**Non-testing**	**22**	**0.930 (93.0%)**	**0.562**
	**Testing**	**8**	**0.852 (85.2%)**	**0.281**
Model-w-3D-tp	Overall	59	0.840 (84.0%)	0.270
	Non-testing	50	0.841 (84.1%)	0.267
	Testing	9	0.833 (83.3%)	0.282
**Model-w-3D-tpi**	**Overall**	**33**	**0.911 (91.1%)**	**0.495**
	**Non-testing**	**23**	**0.927 (92.7%)**	**0.524**
	**Testing**	**10**	**0.815 (81.5%)**	**0.318**

Supplementary Table 9. Hand parameters statistics in each score group in our cohort dataset (MDS-UPDRS item scored by the clinician).

Hand side	MDS-UPDRS item score	Frequency (Hz)		Intensity (arbitrary unit, A.U.)		FI value (A.U./s)		Peak (distance/thumb-length)	
		Average	Median	Average	Median	Average	Median	Average	Median
Left-hand finger taps	0	3.177±0.707	3.157	0.086±0.041	0.081	0.265±0.117	0.253	0.545±0.167	0.521
	1	2.624±0.802	2.589	0.085±0.046	0.074	0.205±0.097	0.196	0.538±0.203	0.499
	2	2.418±0.750	2.420	0.062±0.036	0.054	0.137±0.072	0.129	0.442±0.158	0.426
	3+	1.593±0.432	1.609	0.054±0.028	0.046	0.074±0.030	0.071	0.426±0.127	0.399
Right-hand finger taps	0	3.296±0.927	3.355	0.075±0.037	0.071	0.235±0.114	0.226	0.528±0.152	0.501
	1	2.429±0.739	2.402	0.070±0.040	0.066	0.158±0.081	0.159	0.492±0.151	0.457
	2	2.473±0.931	2.354	0.052±0.031	0.041	0.111±0.065	0.100	0.410±0.144	0.373
	3+	1.521±0.520	1.375	0.039±0.023	0.027	0.048±0.019	0.042	0.374±0.072	0.369

Supplementary Table 10. Performance of the models for predicting the MDS-UPDRS scores in the PDMotorDB dataset (label: original scores).

Model-wo-3D: basic model without 3D rotation; Model-w-3D-t: model with 3D rotation at the training stage only; Model-w-3D-ti: model with 3D rotation at training and inference stage; Model-w-3D-tp: model with 3D rotation at training and model-picking stage; Model-w-3D-tpi: model with 3D rotation at training, model-picking and inference stage; NSE: the number of significant error files; AAC: acceptable accuracy; Kappa: Cohen's kappa coefficient.

Hand side	Model	NSE	AAC	Kappa
Left-hand finger taps	Model-wo-3D	76	0.789 (78.9%)	0.033
	Model-w-3D-t	34	0.906 (90.6%)	0.222
	Model-w-3D-ti	78	0.783 (78.3%)	0.083
	Model-w-3D-tp	23	0.936 (93.6%)	0.152
	Model-w-3D-tpi	88	0.756 (75.6%)	0.058
Right-hand finger taps	Model-wo-3D	21	0.913 (91.3%)	0.024
	Model-w-3D-t	22	0.909 (90.9%)	0.061
	Model-w-3D-ti	17	0.930 (93.0%)	0.073
	Model-w-3D-tp	58	0.760 (76.0%)	0.050
	Model-w-3D-tpi	32	0.868 (86.8%)	0.087
