# Supplementary material for: FastEval Parkinsonism: an instant deep learning–assisted video-based online system for Parkinsonian motor symptom evaluation
Source: NPJ Digit Med. 2024 Feb 8;7:31. doi: 10.1038/s41746-024-01022-x (PMC10853559; doi:10.1038/s41746-024-01022-x)
Supplement: Supplementary file 2 — Reporting Summary [file 41746_2024_1022_MOESM2_ESM.pdf]

nature portfolio

Corresponding author(s): Yufeng Jane Tseng

Last updated by author(s): Jan 15, 2024

Reporting Summary

Nature Portfolio wishes to improve the reproducibility of the work that we publish. This form provides structure for consistency and transparency in reporting. For further information on Nature Portfolio policies, see our Editorial Policies and the Editorial Policy Checklist.

Statistics

For all statistical analyses, confirm that the following items are present in the figure legend, table legend, main text, or Methods section.

n/a	Confirmed	
☐	☒	The exact sample size (*n*) for each experimental group/condition, given as a discrete number and unit of measurement
☒	☐	A statement on whether measurements were taken from distinct samples or whether the same sample was measured repeatedly
☒	☐	The statistical test(s) used AND whether they are one- or two-sided<br>*Only common tests should be described solely by name; describe more complex techniques in the Methods section.*
☒	☐	A description of all covariates tested
☒	☐	A description of any assumptions or corrections, such as tests of normality and adjustment for multiple comparisons
☒	☐	A full description of the statistical parameters including central tendency (e.g. means) or other basic estimates (e.g. regression coefficient) AND variation (e.g. standard deviation) or associated estimates of uncertainty (e.g. confidence intervals)
☒	☐	For null hypothesis testing, the test statistic (e.g. *F*, *t*, *r*) with confidence intervals, effect sizes, degrees of freedom and *P* value noted<br>*Give P values as exact values whenever suitable.*
☒	☐	For Bayesian analysis, information on the choice of priors and Markov chain Monte Carlo settings
☒	☐	For hierarchical and complex designs, identification of the appropriate level for tests and full reporting of outcomes
☒	☐	Estimates of effect sizes (e.g. Cohen's *d*, Pearson's *r*), indicating how they were calculated

Our web collection on statistics for biologists contains articles on many of the points above.

Software and code

Policy information about availability of computer code

Data collection	Custom codes were implemented in Python 3.8.12 with PyTorch 1.11.0 and run on an Ubuntu 18.04 or 22.04 system with NVIDIA GTX 1080 Ti and RTX 3060 Ti.
Data analysis	Custom codes were implemented in Python 3.8.12 with PyTorch 1.11.0 and run on an Ubuntu 18.04 or 22.04 system with NVIDIA GTX 1080 Ti and RTX 3060 Ti.

For manuscripts utilizing custom algorithms or software that are central to the research but not yet described in published literature, software must be made available to editors and reviewers. We strongly encourage code deposition in a community repository (e.g. GitHub). See the Nature Portfolio guidelines for submitting code & software for further information.

Data

Policy information about availability of data

All manuscripts must include a data availability statement. This statement should provide the following information, where applicable:

- Accession codes, unique identifiers, or web links for publicly available datasets
- A description of any restrictions on data availability
- For clinical datasets or third party data, please ensure that the statement adheres to our policy

Due to the patient data privacy policy, the original dataset (video clips) would not be publicly accessed. The de-identified data that support the findings of this study are available from the corresponding author upon reasonable request, with the permission of the institution, and after approval of a proposal.

Research involving human participants, their data, or biological material

Policy information about studies with human participants or human data. See also policy information about sex, gender (identity/presentation), and sexual orientation and race, ethnicity and racism.

Reporting on sex and gender	Sex and gender-based analysis has not been performed since this is a hand motor evaluation study.
Reporting on race, ethnicity, or other socially relevant groupings	This information has not been collected.
Population characteristics	Age: HC: 65.4 +/- 12.9; MPS: 74.8 +/- 6.0; PD: 70.8 +/- 8.2; APD: 59.5 +/- 8.1
Recruitment	Patients and healthy subjects participating in this study were recruited from two hospitals, National Taiwan University Hospital (NTUH) and National Taiwan University Cancer Center (NTUCC), during the period from October 19, 2020, to August 31, 2022.
Ethics oversight	Research Ethics Committees at National Taiwan University Hospital

Note that full information on the approval of the study protocol must also be provided in the manuscript.

Field-specific reporting

Please select the one below that is the best fit for your research. If you are not sure, read the appropriate sections before making your selection.

☐ Life sciences ☒ Behavioural & social sciences ☐ Ecological, evolutionary & environmental sciences

For a reference copy of the document with all sections, see nature.com/documents/nr-reporting-summary-flat.pdf

Behavioural & social sciences study design

All studies must disclose on these points even when the disclosure is negative.

Study description	A quantitative study
Research sample	Patients and healthy subjects participating in this study were recruited from two hospitals, National Taiwan University Hospital (NTUH) and National Taiwan University Cancer Center (NTUCC), during the period from October 19, 2020, to August 31, 2022.
Sampling strategy	210 patients' visits from 186 participants (103 PD, 24 participants with atypical parkinsonism (APD), 47 healthy controls (HCs), 12 elderly with mild parkinsonism signs (MPS)) were enrolled from one community-based populations and two hospital-based cohorts from National Taiwan University Hospital (NTUH) and National Taiwan University Cancer Center (NTUCC).
Data collection	All subjects provided consent for data management and usability prior to data collection. Each subject performed a finger-tapping task twice for each hand, which was recorded by a ZED camera with 720p (width: 720 pixels, height: 1280 pixels) and 60 frames per second (fps) in a side view. Every person continuously performed finger taps for approximately 10 seconds in this study.
Timing	from October 19, 2020, to August 31, 2022.
Data exclusions	No data were excluded
Non-participation	No participants dropped out
Randomization	The non-testing and testing datasets were uniformly randomly divided with a ratio of 0.85 to 0.15 using a Python package, Random, resulting in 362 video clips in the former and 58 clips in the latter dataset for each hand side.

Reporting for specific materials, systems and methods

We require information from authors about some types of materials, experimental systems and methods used in many studies. Here, indicate whether each material, system or method listed is relevant to your study. If you are not sure if a list item applies to your research, read the appropriate section before selecting a response.

Materials & experimental systems

n/a	Involved in the study
☒	☐ Antibodies
☒	☐ Eukaryotic cell lines
☒	☐ Palaeontology and archaeology
☒	☐ Animals and other organisms
☒	☐ Clinical data
☒	☐ Dual use research of concern
☒	☐ Plants

Methods

n/a	Involved in the study
☒	☐ ChIP-seq
☒	☐ Flow cytometry
☒	☐ MRI-based neuroimaging

Plants

Seed stocks	NA
Novel plant genotypes	NA
Authentication	NA
